# Single-Cell Resolution of Uncultured Magnetotactic Bacteria via Fluorescence-Coupled Electron Microscopy

**DOI:** 10.1128/AEM.00409-17

**Published:** 2017-05-31

**Authors:** Jinhua Li, Heng Zhang, Nicolas Menguy, Karim Benzerara, Fuxian Wang, Xiaoting Lin, Zhibao Chen, Yongxin Pan

**Affiliations:** a Key Laboratory of Earth and Planetary Physics, Institute of Geology and Geophysics, Chinese Academy of Sciences, Beijing, People's Republic of China; b Laboratory for Marine Geology, Qingdao National Laboratory for Marine Science and Technology, Qingdao, People's Republic of China; c France-China Biomineralization and Nano-Structures Laboratory, Chinese Academy of Sciences, Beijing, People's Republic of China; d College of Life Science and Technology, Heilongjiang Bayi Agricultural University, Daqing, People's Republic of China; e IMPMC, CNRS UMR 7590, Sorbonne Universités, MNHN, UPMC, IRD UMR 206, Paris, France; University of Bayreuth

**Keywords:** biomineralization, fluorescence-coupled electron microscopy, FISH, magnetotactic bacteria, TEM

## Abstract

Magnetotactic bacteria (MTB) form intracellular chain-assembled nanocrystals of magnetite or greigite termed magnetosomes. The characterization of magnetosome crystals requires electron microscopy due to their nanoscopic sizes. However, electron microscopy does not provide phylogenetic information for MTB. We have developed a strategy for the simultaneous and rapid phylogenetic and biomineralogical characterization of uncultured MTB at the single-cell level. It consists of four steps: (i) enrichment of MTB cells from an environmental sample, (ii) 16S rRNA gene sequencing of MTB, and (iii) fluorescence *in situ* hybridization analyses coordinated with (iv) transmission or scanning electron microscopy of the probe-hybridized cells. The application of this strategy identified a magnetotactic Gammaproteobacteria strain, SHHR-1, from brackish sediments collected from the Shihe River estuary in Qinhuangdao City, China. SHHR-1 magnetosomes are elongated prismatic magnetites which can be idealized as hexagonal prisms. Taxonomic groups of uncultured MTB were also identified in freshwater sediments from Lake Miyun in northern Beijing via this novel coordinated fluorescence and scanning electron microscopy method based on four group-specific rRNA-targeted probes. Our analyses revealed that major magnetotactic taxonomic groups can be accurately determined only with coordinated scanning electron microscopy observations on fluorescently labeled single cells due to limited group coverage and specificity for existing group-specific MTB fluorescence *in situ* hybridization (FISH) probes. Our reported strategy is simple and efficient, offers great promise toward investigating the diversity and biomineralization of MTB, and may also be applied to other functional groups of microorganisms.

**IMPORTANCE** Magnetotactic bacteria (MTB) are phylogenetically diverse and biomineralize morphologically diverse magnetic nanocrystals of magnetite or greigite in intracellular structures termed magnetosomes. However, many uncultured MTB strains have not been phylogenetically identified or structurally investigated at the single-cell level, which limits our comprehensive understanding of the diversity of MTB and their role in biomineralization. We developed a fluorescence-coupled electron microscopy method for the rapid phylogenetic and biomineralogical characterization of uncultured MTB at the single-cell level. Using this novel method, we successfully identified taxonomic groups of several uncultured MTB and one novel magnetotactic Gammaproteobacteria strain, SHHR-1, from natural environments. Our analyses further indicate that strain SHHR-1 forms elongated prismatic magnetites. Our findings provide a promising strategy for the rapid characterization of phylogenetic and biomineralogical properties of uncultured MTB at the single-cell level. Furthermore, due to its simplicity and generalized methodology, this strategy can also be useful in the study of the diversity and biomineralization properties of microbial taxa involved in other mineralization processes.

## INTRODUCTION

Magnetotactic bacteria (MTB) have been a focus of intense study in biomineralization (1–4). They are a phylogenetically, morphologically, and metabolically diverse and large group of prokaryotes. In particular, their diversity, mechanisms of biomineralization, and magnetic properties have been extensively studied since their discovery in the 1960s and have been of great interest in the fields of biology, geology, and materials science (5–11). Moreover, they have drawn some preliminary interest in medical applications due to the use of magnetic nanoparticles in certain treatments, for example, in magnetic hyperthermia (12).

MTB form intracellular single-domain crystals of magnetite (Fe_3_O_4_) or greigite (Fe_3_S_4_) that are surrounded by a lipid bilayer membrane, which together comprise magnetosomes (4, 7, 10). Magnetosomes are generally organized as single or multiple chains, which allow MTB to passively align and actively swim along geomagnetic field lines toward their optimal niches, generally at and just below the oxic-anoxic interface (OAI) in aquatic environments. This process is referred to as magneto-aerotaxis (13, 14). Magnetosomes in MTB can be preserved as magnetofossils, which have been used to retrieve paleomagnetic and paleoenvironmental information from ancient sediments (15–18). In addition, MTB represent natural model bioreactors, which can be mimicked to produce well-tailored magnetic nanoparticles with enhanced magnetic properties (19, 20).

Molecular biology and microscopy have revealed a significant phylogenetic diversity of MTB and a broad morphological diversity of their magnetosome crystals (8, 10). Most cultured and uncultured MTB are phylogenetically affiliated with the Alphaproteobacteria, Deltaproteobacteria, Gammaproteobacteria, and Nitrospirae (4). Recently, phylogenetic and ultrastructural analyses of micromanipulated single cells collected from sediments of Lake Chiemsee in Upper Bavaria (Germany) detected a large ovoid cell, SKK-01, that belonged to the proposed phylum “Candidatus Omnitrophica” (formerly the candidate division OP3) (21, 22). Previous studies have shown that the numbers, sizes, crystal habits, and chain configurations of magnetosomes are diverse among species and even individual strains (8, 10). However, magnetosome biomineralization has only been studied within a few cultured and uncultured MTB strains. Many uncultured MTB strains have not been phylogenetically identified or structurally investigated at the single-cell level. This represents a key knowledge gap in our understanding of MTB biomineralization and the corresponding relationship between magnetosomes and bacterial species or strains. There is therefore a need for a method to rapidly characterize both the phylogenetic and biomineralogical properties of uncultured MTB at the single-cell level. Such methodologies would enable an accurate assessment of the diversity and mechanisms of biomineralization among MTB from various natural environments.

Fluorescence *in situ* hybridization (FISH) has been widely used for the phylogenetic identification of single microbial cells or microbial populations in complex environments (23–27). This approach has been applied to MTB populations from various freshwater and marine sediments (28–35). Significant advancements in both the spatial and energy resolution of transmission electron microscopy (TEM) over the past decade have provided a powerful platform for obtaining structural, compositional, and magnetic information of MTB at the atomic scale (3, 36, 37). For example, Li et al. recently analyzed the structural and magnetic properties of bullet-shaped magnetosomes in the uncultured “Candidatus Magnetobacterium casensis” strain MYR-1 using various advanced TEM techniques. Magnetosome crystal growth in this bacterium was found to occur in several steps: (i) an initial isotopic growth-forming cuboctahedron (less than approximately 40 nm), followed by (ii) anisotropic growth up to ∼60 to 80 nm and mostly along the [112], [114], or [111] direction, and finally, (iii) kinking and continuous growth along the [001] direction (38).

Recently, correlative fluorescence light and electron microscopy has raised great interest in the life sciences because it combines the specificity of fluorescence labeling with the high structural resolution and cellular environment by the scanning electron microscopy (SEM) or TEM (39). The general and most widespread fluorescence targeting methods use genetically encoded labeling with green fluorescent proteins, immunofluorescent labeling with Nanogold, or a combination of both (40, 41). Oligonucleotide probes directly labeled with Nanogold or Nanogold-labeled antibodies that targeted oligonucleotide probes with fluorescein or digoxigenin have also been used to link the ultrastructure of microbial cells with their phylogenetic affiliations (42–44). Technically, today's approaches generally involve complex sample preparation procedures due to the need for either thin sections or frozen samples for TEM characterization (43, 44).

Here, we developed a strategy for the rapid phylogenetic and biomineralogical characterization of uncultured MTB at the single-cell level by coordinating fluorescence and electron microscopy. Using this method, we identified a novel magnetotactic Gammaproteobacteria strain in brackish sediments collected from the Shihe River estuary in Qinhuangdao City in eastern China (SHHR-1). The biomineralization features of SHHR-1 cells and crystal habits of SHHR-1 magnetosomes were investigated in detail via various advanced TEM approaches. Moreover, we identified several taxonomic groups of uncultured MTB in freshwater sediments collected from Lake Miyun in north Beijing in China using fluorescence-coupled SEM observations on enriched MTB cells that were hybridized with four group-specific rRNA-targeted probes. Our results demonstrate that the fluorescence-coupled electron microscopy method is a simple and efficient strategy that allows for the rapid characterization of phylogenetic and biomineralogical properties of uncultured MTB at the single-cell level. Thus, this strategy offers great promise toward investigating the diversity and biomineralization of natural MTB from various environments, and it may also be applied to other mineralizing functional groups of microorganisms.

## RESULTS

### Identification of SHHR-1 by coupled FISH-SEM and FISH-TEM.

**FIG 1:**
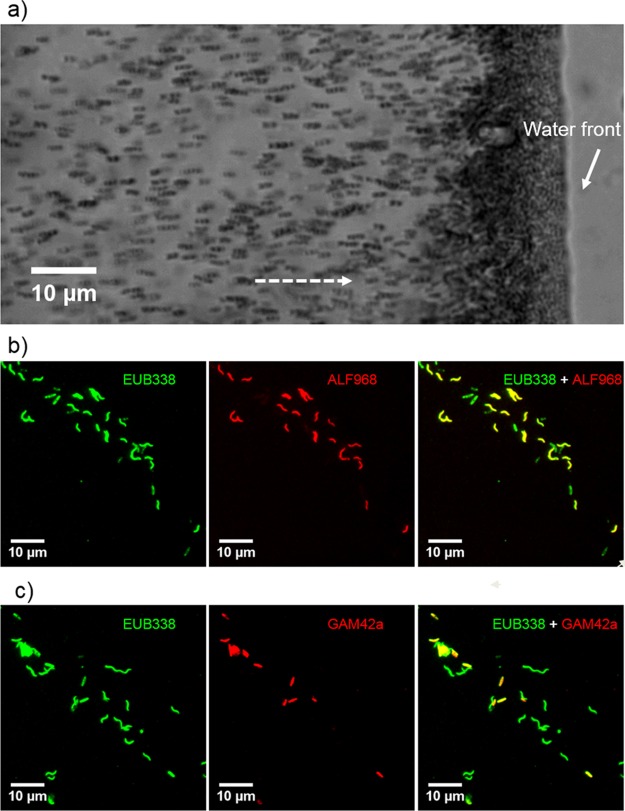
Morphological and FISH identification of SHHR-1 cells. (a) Optical microscopy image of living SHHR-1 cells. Cells are swimming out from one small drop of sediment on the left (photograph not shown) along the applied magnetic field lines (dashed-line arrow) and gathering on the edge of the water droplets. (b) Fluorescence microscopy images of SHHR-1 cells *in situ* hybridized with the 5′-FAM-labeled universal bacterial probe EUB338 and the 5′-Cy3-labeled Alphaproteobacteria-specific probe ALF968. (c) Fluorescence microscopy images of SHHR-1 cells *in situ* hybridized with the 5′-FAM-labeled universal bacterial probe EUB338 and the 5′-Cy3-labeled Gammaproteobacteria-specific probe GAM42a. For each FISH identification, the same microscopy field is shown. Results of hybridization with the EUB338 probe are shown in green and in red for the group-specific probes ALF968 and GMA42a, respectively. Overlapped fluorescence microscopy images are shown for EUB338 plus ALF968 and EUB338 plus GAM42a. Inner-control AMB-1 cells were targeted by both the EUB338 and ALF968 probes. SHHR-1 cells were targeted by the GAM42a probe and the EUB338 probe.

Live MTB cells were magnetically extracted using previously described methods from a sediment microcosm in which the dominant MTB are a group of small rod-shaped bacteria (i.e., SHHR-1). In order to characterize the SHHR-1 taxonomic group, we identified SHHR-1 cells with FISH using four group-specific rRNA-targeted probes (Table 1). Fluorescent labeling of SHHR-1 cells and inner-control Magnetospirillum magneticum strain AMB-1 cells were positively bound by the universal bacterial probe EUB338 but negative for the SRB385Db and BaP probes (Fig. 1 and S1). AMB-1 cells were specifically targeted by the ALF968 probe because of their phylogenetic affiliation with the Alphaproteobacteria (Fig. 1b). In contrast, SHHR-1 cells were labeled only by the GAM42a probe but not by the other three group-specific probes (Fig. 1c and S1), indicating that SHHR-1 likely belongs to the Gammaproteobacteria class.

**FIG 2:**
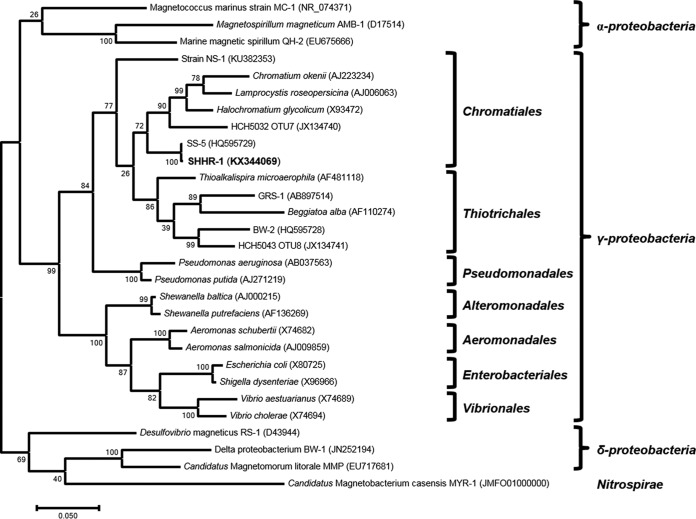
Phylogenetic tree of 16S rRNA gene sequences. Bootstrap values at nodes are given as percentages of 1,000 replicates. GenBank accession numbers are given in parentheses. Scale bar represents 5% sequence divergence.

We then amplified 16S rRNA genes from a sample containing highly magnetically concentrated SHHR-1 cells using universal bacterial primers and constructed a clone library from the 16S rRNA gene amplicons. Thirteen of the 19 sequenced clones contained identical near-full-length 16S rRNA gene sequences. Phylogenetic analysis of 16S rRNA gene sequences indicated that SHHR-1 belongs to the Gammaproteobacteria class of the Proteobacteria phylum and was most closely related to the order Chromatiales (Fig. 2). SHHR-1 16S rRNA gene sequences shared ∼99% nucleotide (nt) sequence identity with the magnetotactic Gammaproteobacteria strain SS-5 (45). Thus, SHHR-1 may represent the same bacterial species as strain SS-5 but may represent a novel magnetotactic Gammaproteobacteria strain in brackish sediments collected from the Shihe River estuary in Qinhuangdao City in eastern China.

**FIG 3:**
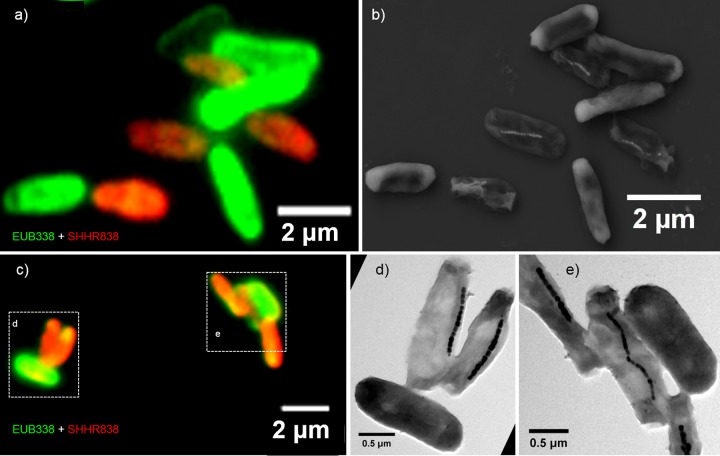
Fluorescence-coupled electron microscopy identification of SHHR-1 cells. (a) Overlapping fluorescence microscopy image of SHHR-1 and E. coli cells mounted on the surface of a cover slide glass and *in situ* hybridized with the 5′-FAM-labeled universal bacterial probe EUB338 (green) and the 5′-Cy3-labeled SHHR838 probe (red). (b) Coordinated SEM image of the same field as in panel a. (c) Overlapping fluorescence microscopy image of SHHR-1 and E. coli cells mounted on the surface of a TEM grid and *in situ* hybridized with the 5′-FAM-labeled universal bacterial probe EUB338 (green) and the 5′-Cy3-labeled SHHR838 probe (red). (d) Coordinated TEM image of the same field indicated by dashed-line box (left) as in panel c. (e) Coordinated TEM image of the same field indicated by dashed-line box (right) as in panel c. Those bacteria that are only fluorescently labeled with the EUB338 probe and do not contain magnetosomes are inner-control E. coli cells. In contrast, those bacteria that are fluorescently labeled with both the EUB338 and SHHR838 probes (yellow-red colors) and contain magnetosomes are SHHR-1 cells.

We then performed coordinated FISH-SEM and FISH-TEM analyses using a 16S rRNA-specific probe for the SHHR-1 phylogenetic group (SHHR838; Table 1). Since the same sample had fluorescence microscopy data coupled with electron microscopy, both the phylogenetic affiliation and nanometer-scale structural features of each single cell(s) could be determined. The SHHR838 probe specifically targeted SHHR-1 cells but did not label Escherichia coli control cells. All bacterial cells that were positively labeled by both the 5′-FAM-labeled (FAM, 6-carboxyfluorescein) universal bacterial probe EUB338 (green) and the 5′-Cy3-labeled SHHR-1-specific probe SHHR838 (red) contained magnetosomes. All of the cells that were labeled only by the EUB338 (green) probe (E. coli control cells) failed to exhibit any magnetosomes (Fig. 3, S2, and S3). These results confirmed that the 16S rRNA gene sequence originated from the SHHR-1 strain and that the strain is a magnetotactic gammaproteobacterium.

### Morphological features and crystal habits of SHHR-1 magnetosomes.

**FIG 4:**
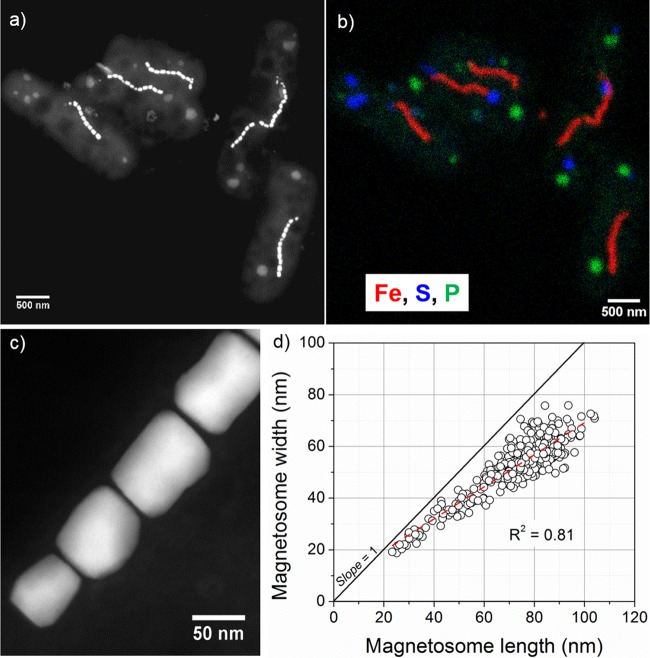
Morphological and chemical features of SHHR-1 cells. (a) HAADF-STEM image of five SHHR-1 cells. (b) Chemical composition map of the same five SHHR-1 cells as shown in panel a. HAADF-STEM imaging and STEM-EDXS mapping analyses show that SHHR-1 cells contain magnetite-type magnetosomes as single chains, as well as irregular polyphosphate and sulfur-rich inclusions. (c) High-magnification HAADF-STEM image of SHHR-1 magnetosomes showing their prismatic shapes and chain alignment along the long axes of individual particles. (d) Plot of crystal length versus width showing a linear relationship between crystal length and width of SHHR-1 magnetosomes.

TEM observations indicated that SHHR-1 cells are rod-shaped, with an average cell length of 2.5 μm with a standard deviation of 0.5 μm and a diameter of 0.9 μm with a standard deviation of 0.07 μm (*n* = 52). SHHR-1 possesses a single polar flagellum and contains an average of 15 magnetosomes per cell (*n* = 142; with a standard deviation of 4 magnetosomes per cell). Magnetosomes are organized as single chains aligned parallel to the long axis of the cells (Fig. 4a and S4a to f). In addition to magnetosomes, SHHR-1 cells typically contain several intracellular granules that are irregular in shape and diverse in size, ranging from several nanometers to several hundreds of nanometers (Fig. 4a and S4b). Energy-dispersive X-ray spectroscopy (EDXS) elemental mapping operating in the high-angle annular dark-field scanning transmission electron microscopy (HAADF-STEM) mode showed that the magnetosomes are rich in Fe and O, suggesting that they are magnetite. Other intracellular granules could be divided into two groups based on their chemical compositions: some were rich in sulfur, while others were rich in phosphorus, calcium, and oxygen, indicating that they were sulfur and polyphosphate inclusions, respectively (Fig. 4b and S5).

SHHR-1 magnetosomes have elongated prismatic shapes, with an average crystal length of 72.9 nm with a standard deviation of 15.7 nm, a diameter of 52.6 nm with a standard deviation of 11.0 nm, and a shape factor of 0.73 with a standard deviation of 0.07 (*n* = 423) (Fig. 4c and S4g to i). SHHR-1 magnetosomes exhibited a crystal size distribution and growth mode typical of prismatic and cuboctahedral magnetite magnetosomes (46, 47). This was indicated by crystal length and width distributions that were negatively skewed and exhibited a nearly constant width-to-length ratio (Fig. 4d and S4g to i). These results suggest that magnetosomes within SHHR-1 grow by a mechanism similar to that of known magnetotactic Alphaproteobacteria, i.e., homothetic growth within magnetosome membranes (46, 48).

**FIG 5:**
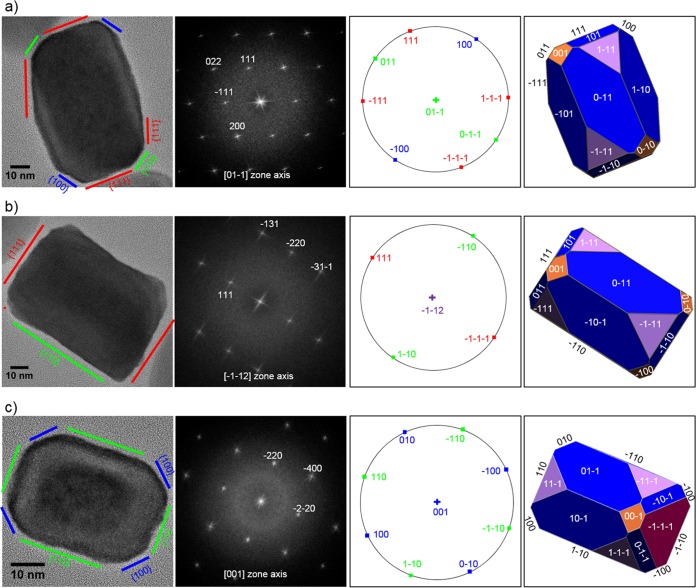
HRTEM images of three representative magnetosomes recorded along different zone axes (first column), their corresponding indexed fast Fourier transform (FFT) patterns (second column), stereographic projections (third column), and morphological models (fourth column) oriented with respective to the FFT and HRTEM images. The outlines and lattice fringes of the magnetosomes in their HRTEM images are consistent with prismatic models. (a) For the first particle imaged along the [01-1] zone axis, four well-developed {111} faces can be identified at both ends and sides of the prism, and two small {100} and {110} faces were identified at the corners of the prism. (b) For the second particle imaged along the [-1-12] zone axis, two large {111} and {110} faces can be observed to roughly terminate at both the ends and sides of the prism. (c) The third particle recorded along the [001] zone axis appears to be capped and terminated by {110} faces at both the ends and sides and truncated by four small {100} faces at the corners.

The morphologies of 11 individual SHHR-1 magnetosomes from two cells were investigated using high-resolution TEM (HRTEM) analyses (HRTEM images in the supplemental material). Assuming near-uniform magnetosome morphology with only minor differences, we derived an idealized crystal habit that can explain the observed two-dimensional (2D) projections deduced from HRTEM images. These observations allowed reconstruction of the crystal morphology of the SHHR-1 magnetosomes based on {111}, {110}, and {100} forms, i.e., a hexagonal prism with six large {110} faces, capped by two large {111} end faces and truncated by six small {110}, six small {100}, and six small {111} end faces (Fig. 5). Although other possibilities could exist, this idealized crystal morphology explains most HRTEM images of mature magnetosomes obtained in this study. Moreover, this crystal morphology reveals that the crystals of SHHR-1 magnetosomes are hexagonal prisms elongated and aligned along one of the 〈111〉 directions of magnetite (see HRTEM images in the supplemental material).

### Coordinated FISH-SEM identification of uncultured MTB from Lake Miyun.

**FIG 6:**
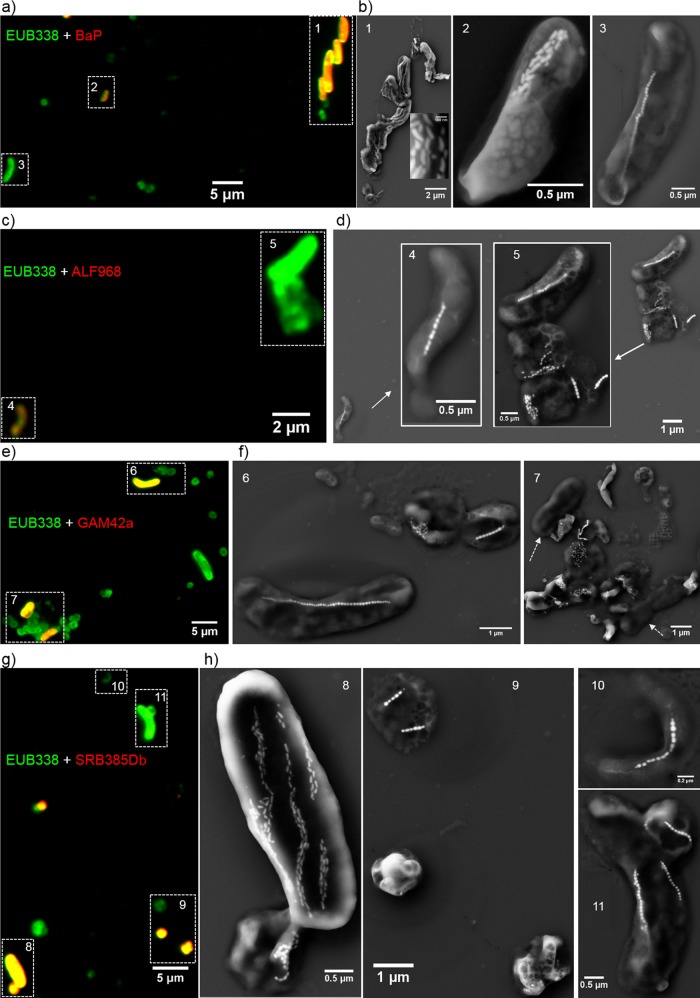
Coupled FISH-SEM identifications of uncultured MTB in freshwater sediments collected from Lake Miyun in north Beijing, China. (a) Overlapping fluorescence microscopy image of Miyun MTB cells *in situ* hybridized with the 5′-FAM-labeled universal bacterial probe EUB338 (green) and the 5′-Cy3-labeled BaP probe (red). (b) High-magnification SEM images of the same cell(s) indicated by numbers in panel a. (c) Overlapped fluorescence microscopy image of Miyun MTB cells hybridized with the 5′-FAM-labeled universal bacterial probe EUB338 (green) and the 5′-Cy3-labeled Alphaproteobacteria-specific probe ALF968 (red). (d) High-magnification SEM images of the same cell(s) indicated by numbers in panel c. (e) Overlapped fluorescence microscopy image of Miyun MTB cells *in situ* hybridized with the 5′-FAM-labeled universal bacterial probe EUB338 (green) and the 5′-Cy3-labeled Gammaproteobacteria-specific probe GAM42a (red). (f) High-magnification SEM images of the same cell(s) indicated by numbers in panel e. (g) Overlapped fluorescence microscopy image of Miyun MTB cells *in situ* hybridized with the 5′-FAM-labeled universal bacterial probe EUB338 (green) and the 5′-Cy3-labeled Desulfobacteraceae-specific probe SRB385Db (red). (h) High-magnification SEM images of the same cell(s) indicated by numbers in panel g.

In order to test the ability of the coupled FISH-SEM approach to identify taxonomic groups and magnetosome types of uncultured MTB, we applied our methods to uncultured MTB cells from a more complex and diverse environment in sediments collected from Lake Miyun in north Beijing, China. Miyun MTB are phylogenetically and morphologically diverse (49). However, none of these MTB have been phylogenetically and morphologically characterized using the coupled FISH-SEM method; as a result, the relationship between magnetotactic taxonomic groups and biomineralization features has not been understood. As expected, all MTB cells from Lake Miyun were fluorescently labeled by the 5′-FAM-labeled universal bacterial probe EUB338 (green) (Fig. 6 and S6 to S9). The MTB community was dominated by magnetotactic Alphaproteobacteria and Nitrospirae, which is consistent with previous phylogenetic analyses (49). Coordinated FISH-SEM analyses indicated that the 5′-Cy3-labeled BaP probe (red) specifically targeted two magnetotactic Nitrospirae strains: giant rod-shaped MYR-1 (i.e., “Candidatus Magnetobacterium casensis”) and watermelon-shaped MWB-1 (Fig. 6a and b and S6). One small rod-shaped bacterium (tentatively named MYR-2) was also fluorescently labeled by the BaP probe. Coordinated SEM observations indicated that this bacterium contains one bundle of bullet-shaped magnetosomes (Fig. 6a and b and S6). Interestingly, the ALF968 probe targeted only two types of helical MTB that both produced cuboctahedral or prismatic magnetosome crystals and some nonmagnetotactic bacterial cells. Diverse magnetotactic cocci formed single, double, and quadruple chains of prismatic magnetosomes, dispersed aggregates, or clusters within the cells. They were not fluorescently labeled by the 5′-Cy3-labeled Alphaproteobacteria-specific probe ALF968 (red) (Fig. 6c and d and S7). The 5′-Cy3-labeled Gammaproteobacteria-specific probe GAM42a did not target the magnetotactic Nitrospirae, spirilla, or cocci. In contrast, one relatively large rod-shaped bacterium (tentatively named MYR-3) that comprised a single chain of cuboctahedral magnetosomes was labeled by the GAM42a probe (Fig. 6e and f and S8) but not by the three other probes (Fig. 6, S6, S7, and S9). Some nonmagnetotactic bacteria were also fluorescently labeled by the GAM42a probe (Fig. 6e and f and S8). Surprisingly, strain MYR-1 and one small magnetotactic coccus, in which magnetosomes were arranged as dispersed aggregates or clusters, were also fluorescently labeled by the 5′-Cy3-labeled Desulfobacteraceae-specific probe SRB385Db, while other MTB cells were fluorescently labeled by the EUB338 probe only (Fig. 6g and h and S9).

## DISCUSSION

### Fluorescence-coupled electron microscopy as a novel tool to study MTB at the single-cell level.

Here, we describe a new strategy that allows the rapid phylogenetic and biomineralogical study of single MTB cells by coupled rRNA-targeting FISH and SEM/TEM analyses. Our strategy consists of four steps: (i) the enrichment of uncultured MTB cells from an environmental sample, (ii) 16S rRNA gene sequencing of MTB, (iii) FISH with fluorescently labeled rRNA-targeted oligonucleotide probes and fluorescence microscopy observations, and (iv) coordinated SEM and TEM analyses on probe-hybridized MTB cells. The coordinated observations are enhanced by targeting specific areas, such as the edge of the sample on the cover glass or the center position of the TEM grid. Our strategy enables fluorescence and electron microscopy observations to be made on chemically immobilized and ethanol-dehydrated cells. Experimental results show that the cell morphology is weakly modified and easily recognized by subsequent SEM or TEM observations after a standard procedure in FISH studies and a vacuum pretreatment (Fig. 4). It does not provide fluorescent and structural information from viable cells and ultrathin sections of cells. However, our method has the advantage of directly linking each MTB 16S rRNA genotype to an important phenotypical feature of MTB (i.e., magnetosome morphology) using high-specificity oligonucleotide probes and leveraging the high spatial resolutions of SEM and TEM. Furthermore, our approach is relatively easy to achieve through routine sample preparation procedures and the use of conventional fluorescence and electron microscopes, making it feasible to apply across standard laboratory settings. The approach can be conducted even when a special SEM equipped with a fluorescence digital camera or a dedicated TEM equipped with an *in situ* fluid cell TEM holder is unavailable (41, 50, 51).

Spring et al. (43) developed a method for linking the ultrastructure of enriched MTB cells with their 16S rRNA sequence via TEM of ultrathin sections that were hybridized *in situ* with digoxigenin- and fluorescein-labeled polynucleotide probes. The bound polynucleotide probe was detected by high-resolution TEM after incubation of sections with gold-labeled antibodies that were specific for fluorescein or digoxigenin. Using this method, the authors successfully found one ovoid MTB from the Itaipu Lagoon of Rio de Janeiro, Brazil, that exhibits an Itaipu I 16S rRNA genotype and forms unusually large magnetosomes. More recently, Woehl et al. (51) successfully imaged cells and magnetosomes of Magnetospirillum magneticum AMB-1 in liquid using a correlative STEM and fluorescence microscopy methodology. These previous studies made valuable contributions toward identifying MTB cells and studying magnetosome biomineralization at the single-cell level. However, both methods involved relatively complex procedures of sample preparation or special equipment, which therefore limit their application.

The coupled FISH-SEM approach described here is carried out by mounting probe-hybridized cells on a cover slide glass surface, which is also suitable for coordinated Raman microspectroscopy and NanoSIMS analyses (52, 53). Coupled FISH-TEM is performed by mounting probe-hybridized cells on the carbon film of a TEM grid, which can also be used for coordinated analysis by synchrotron-based scanning transmission X-ray microscopy (54). Therefore, both coupled FISH-SEM and FISH-TEM approaches may also be combined with other microscopy and spectromicroscopy methods to provide phylogenetic, morphological, structural, mineralogical, compositional, and isotopic information on single cells at the nano- and even atomic-scale levels. Due to its simplicity and applicability to other biomineralization processes, the strategy developed here can also be useful for understanding the diversity and biomineralization processes in microbial taxa other than MTB.

### Applications and importance of fluorescence-coupled electron microscopy.

Owing to the limitations of resolution in conventional optical microscopy (∼0.2 μm), fluorescence microscopic observations alone cannot identify MTB taxa from a mixed-microbe sample. Although bacterial cells labeled by certain group-specific rRNA-targeted probes are detected via fluorescence microscopy (Fig. 6), SEM observations were needed to accurately determine their taxonomic groups.

First, we used the coupled FISH-SEM method to identify Nitrospirae MTB using the BaP probe in freshwater sediments from Lake Miyun. The BaP probe was primarily designed to target “Candidatus Magnetobacterium bavaricum” (55). Nitrospirae MTB strains MYR-1, MWB-1, and MY3-5B were first detected in Miyun sediments and later also observed in freshwater sediments collected from Lake Beihai in Beijing (30, 31). In this study, strains MYR-1 and MWB-1 were successfully distinguished from other MTB cells by our coupled FISH-SEM method, i.e., positive FISH tests and unusual structural features (giant rod cell morphology for MYR-1 and watermelon cell morphology for MWB-1, and bullet-shaped magnetosomes arranged in several chains for both) (Fig. 6a and b and S6). Strain MY3-5B, a small coccoid-to-ovoid bacterium that forms bullet-shaped magnetosomes arranged in several chains (31), was not found in this study, possibly due to low population abundances. In contrast, strain MYR-2, a small rod-shaped cell that forms a bundle of bullet-shaped magnetosomes, was detected. MYR-2 is morphologically similar to strain MHB-1 that was detected from lake sediments in Bremen (Waller See, northern Germany) and which is phylogenetically affiliated with the Nitrospirae phylum (33). Therefore, MYR-2 may represent a novel species or strain belonging to the Nitrospirae phylum inhabiting Lake Miyun and warrants further phylogenetic and biomineralogical analyses.

Second, we identified Alphaproteobacteria MTB using the ALF968 probe that was originally designed to target Alphaproteobacteria (56). Two types of helical MTB were fluorescently labeled and morphologically identified by our coupled FISH-SEM analysis (Fig. 6c and d and S7). Almost all magnetotactic cocci failed to be fluorescently labeled by the ALF968 probe. Coordinated SEM observations revealed that those cocci generally formed prismatic magnetosomes that were arranged into single, double, and quadruple chains, or into dispersed aggregates or clusters within the cells. We then analyzed the group coverage and specificity of the ALF968 probe on almost all available high-quality full-length 16S rRNA sequences of MTB from the NCBI GenBank database (*n* = 421) (available at https://www.ncbi.nlm.nih.gov/). Our analyses suggest that ALF968 can target most Magnetospirillum species, but almost all cultured and uncultured magnetotactic cocci of the order Magnetococcales in the Alphaproteobacteria class cannot hybridize to the ALF968 probe. This is due to a single guanine (G) mismatch at position 979 in the ALF968 probe and a corresponding adenine (A) in the unmatched target sequences. In addition, optimization of FISH conditions by using the ALF968 probe at 20% formamide, which has been proven to yield a better fluorescence signal (56), is worth a try to test its effectiveness. Alternatively, a new probe, FMTCf (5′-TAAAGCCCTTTYAGTGGGAA-3′, positions 431 to 450), which was used as the forward primer specific for amplifying freshwater alphaproteobacterial magnetotactic cocci (57), may be more effective in detecting uncultured alphaproteobacterial magnetotactic cocci.

Finally, we tested for the presence of deltaproteobacterial and gammaproteobacterial MTB from surface sediments of Lake Miyun with the coupled FISH-SEM method, although they have not previously been found there. The SRB385Db probe was designed to target members of the Desulfovibrionaceae family of the Deltaproteobacteria (58). It can hybridize with most magnetotactic Deltaproteobacteria, which are all sulfate reducers. Interestingly, SRB385Db also hybridizes with the Nitrospirae bacterium “Candidatus Magnetobacterium casensis” strain MYR-1 (Fig. 6g and h and S9). This misidentification is likely due to the thymine (T) at position 390 in the SRM385Db probe weakly binding to the guanine (G) in the target sequence. Based on this result, we assumed that SRB385Db could target Desulfovibrio magneticus strains RS-1 (accession no. NR027575) (59) and FH-1 (accession no. JF330268) (10) because of possible T-G bond formation during hybridization. Such a compromise in base pairing (e.g., T-G bond or A-C) increases the group coverage of the probe, but it also results in outgroup hits, as we have shown.

In addition, we found that strain MYR-3, a large rod-shaped bacterium that forms a single chain of cuboctahedral magnetosomes, was specifically targeted by the GAM42a probe rather than by the BaP, ALF968, or SRM385Db probe (Fig. 6 and S8). Due to possible false-positive identifications (24, 60), the GAM42a probe alone does not provide unambiguous identification of magnetotactic Gammaproteobacteria. Therefore, we still cannot conclude whether strain MYR-3 belongs to the magnetotactic Gammaproteobacteria or Betaproteobacteria without simultaneously using the 23S rRNA-targeted probes GAM42a and BET42a or sequencing its 16S rRNA gene.

### Identification and biomineralization of Gammaproteobacteria strain SHHR-1.

Using coupled FISH-SEM and FISH-TEM, we successfully identified SHHR-1 in brackish sediments collected from the Shihe River estuary in Qinhuangdao City, eastern China. SHHR-1 is phylogenetically affiliated with the Gammaproteobacteria class of the Proteobacteria phylum. In contrast to a large number of MTB species or strains discovered in the Alphaproteobacteria and Deltaproteobacteria, only six MTB strains have been recently identified that belong to the Gammaproteobacteria class. Of these six Gammaproteobacteria strains, two axenic cultures were isolated from sediments collected from the Badwater Basin in Death Valley National Park and the Salton Sea in California, USA (45), and two uncultured MTB operational taxonomic unit 7 (OTU7) and OTU8 strains were detected in the city moat of Xi'an, China (29); another unusually large uncultured rod-shaped Gammaproteobacteria species was detected from a shallow freshwater pond in Kanazawa, Japan (28), and one uncultured vibrioid gammaproteobacterium was collected from a brackish lagoon in Brazil (61). However, gammaproteobacterial biomineralization of magnetosomes has yet to be studied.

TEM analyses indicated that strain SHHR-1 is morphologically nearly identical to strain SS-5 in terms of cell morphology, size, the properties of its magnetosome chain, and the presence of a single polar flagellum and polyphosphate inclusions (45). It has been suggested that magnetosome crystals in strain SS-5 were slightly distorted octahedrons (45). However, HRTEM observations performed on dozens of individual magnetosomes coupled with morphological modeling demonstrated that strain SHHR-1 forms elongated prismatic magnetites. This difference in crystal habits between magnetosomes of strains SS-5 and SHHR-1 may reflect a biogeographical effect on magnetosome biomineralization. It is also possible that the cultivation of strain SS-5 in an artificial growth medium changes the magnetosome shape. Alternatively, standard TEM observations generally provide a bright-field image of a crystal outline, much like a shadow (62, 63). Thus, it is difficult to distinguish a crystal edge from a crystal face; therefore, this perceived difference may arise from inaccurate reconstruction of crystal habits from a few two-dimensional TEM images. It is therefore possible that strain SS-5 produces magnetosome crystals with a hexagonal prismatic habit that was not accurately determined by TEM images alone (45). In order to assess whether the crystal habits of magnetosomes are species/strain-specific and/or affected by the environment, additional fluorescence-coupled electron microscopy studies on uncultured MTB cells at the single-cell level, as well as systematic TEM studies of magnetosome biomineralization, are necessary. Such studies that link the phylogenetic identity of each MTB with the crystal habits of their magnetosomes are critical for better understanding the phylogenetic diversity of MTB and mechanisms of magnetosome biomineralization.

## MATERIALS AND METHODS

### Sediment sampling, microcosm design, collection of MTB, and sample preparation.

Water and sediment samples were collected in the estuary of the Shihe River in Qinhuangdao City, eastern China. The sampling site is located inside the estuary (39°57′55.6″N, 119°47′9.4″E) and is a brackish lacustrine environment. The site was characterized by a salinity of ∼23.9 ppt, pH 7.5, and a temperature of 19°C at the time of sampling (July 2015).

Sediment samples (∼10-cm depth) and surface water were collected at a water depth of ∼1 to 2 m near the shore. One-liter plastic bottles were filled to about 60% of their volume with sediments, and the other 40% with was filled with water that overlaid the sediments. Once in the laboratory, microcosms were set up by incubating samples in darkness at ambient temperature (∼20°C). MTB in the microcosms were routinely checked using the hanging-drop technique (64), using an Olympus microscope (BX51) equipped with a phase-contrast, fluorescence, and DP70 digital camera system (Olympus Corp., Tokyo, Japan).

After several weeks of incubation in the laboratory, one microcosm was found to be dominated by a group of small rod-shaped bacteria (i.e., SHHR-1) that swam toward the south pole of a bar magnet (North-seeking MTB) (Fig. 1a). Living MTB cells were magnetically extracted from the microcosm using a homemade magnetic separation apparatus, as previously described (65). MTB cells collected from about 200 ml of MTB-rich slurry were concentrated in a 1.5-ml Eppendorf tube, washed three times with distilled water, and finally resuspended in ∼100 μl of Milli-Q water for additional experiments. A sufficient amount of clean SHHR-1 cells was successfully extracted from sediments for subsequent experiments.

About 5 μl of SHHR-1 cells was used for TEM analyses. A small drop of cells (∼1 to 2 μl) was deposited on a carbon-coated TEM grid and incubated for about 1 h. The grid was then washed three times with Milli-Q water, dried within an anaerobic chamber ([O_2_], <300 ppm; Coy Labs, USA), and finally maintained in a pure N_2_ atmosphere prior to TEM observations. About 20 μl of SHHR-1 cells was boiled for 10 min and stored at −20°C for PCR amplification of 16S rRNA genes. Finally, about 75 μl of MTB cells was centrifuged and resuspended in 75 μl of 0.22-μm-pore filtered phosphate-buffered saline (PBS) buffer (0.1 M PBS [pH 7.4]). Twenty-five microliters of 4% paraformaldehyde was added for fixation at 4°C. After 12 h of fixation, the SHHR-1 cells were centrifuged, suspended in a mixed solution of 200 μl of 0.1 M PBS buffer (pH 7.4) and 200 μl ethanol (100%), and finally stored at −20°C for FISH coordinated with SEM and TEM analyses.

### PCR amplification, 16S rRNA gene sequencing, and phylogenetic analyses.

16S rRNA genes were amplified using the universal bacterial primers 27F (5′-AGAGTTTGATCCTGGCTCAG-3′) and 1492R (5′-GGTTACCTTGTTACGACTT-3′) (66), as described previously (49). Briefly, each 50-μl PCR mixture contained 1 μl of template, 25 μl of DreamTaq PCR master mix (MBI Fermentas), 2 μl of each primer (10 μM), and 20 μl of Milli-Q water. The PCR conditions were 95°C for 3 min, 30 cycles at 95°C for 1 min, 55°C for 1.5 min, and 72°C for 1.5 min, and a final 10-min extension at 72°C. To avoid potential amplification biases, triplicate PCR products for each sample were pooled and purified using 0.8% (wt/vol) agarose gel electrophoresis (described below). All PCR controls without added template were negative.

PCR products were purified using an E.Z.N.A. gel extraction kit (Omega Bio-tek, Inc. USA), ligated with the pMD19-T vector (TaKaRa, Japan), and cloned in Escherichia coli (strain DH5α) competent cells (Tiangen, Beijing, China), according to the manufacturer's instructions. Thirty clones were randomly picked and sequenced using the vector primers M13-47 (5′-CGCCAGGGTTTTCCCAGTCACGAC-3′) and RV-M (5′-GAGCGGATAACAATTTCACACAGG-3′) at the Huada Genome Center (Beijing, China).

In this study, 13 clone sequences were analyzed after discarding sequences of insufficient length or low quality. The lengths of the sequences were about 1,400 to 1,500 bp, covering nearly the full length of the 16S rRNA gene. The sequences were compared against the NCBI GenBank database using the BLASTN algorithm to identify previously published 16S rRNA gene sequences with high nucleotide identity. Multiple-sequence alignments were performed using the ClustalW algorithm with manual correction (67). Maximum likelihood phylogenetic analysis was conducted using the alignments in the MEGA software package (version 7.0) (68). Bootstrap values were calculated with 100 replicates.

### Coupled FISH-SEM and FISH-TEM analyses.

Four group-specific rRNA-targeted probes, ALF968 (5′-GGTAAGGTTCTGCGCGTT-3′) (56), GAM42a (5′-GCCTTCCCACATCGTTT-3′) (60), SRB385Db (5′-CGGCGTTGCTGCGTCAGG-3′) (58), and BaP (5′-GCCATCCCCTCGCTTACT-3′) (31, 33, 55), were used to identify the phylogenetic groups of MTB obtained in this study. Additionally, we designed the probe SHHR838 (5′-ACCCTTTTATGAGTCCAACGGCT-3′, positions 838 to 860) to specifically target 16S rRNA gene sequences of SHHR-1-like cells (Table 1). Probe specificity was evaluated by using the online probe evaluation tools probeCheck and probeBase (69, 70). Except for magnetotactic Gammaproteobacteria strain SS-5, no other sequence in the SILVA111 database has a matching complementary sequence (the minimum number of mismatches is 2). The calculated melting temperature (*T_m_*) of the probe SHHR838 is about 63°C (salt adjusted) (71). In this study, the universal bacterial probe EUB338 (5′-GCTGCCTCCCGTAGGAGT-3′) was used as a positive-control probe of bacteria for FISH (26). Probe EUB338 was synthesized and fluorescently labeled with fluorescein phosphoramidite FAM at the 5′ end. All other probes were synthesized and fluorescently labeled with the hydrophilic sulfoindocyanine dye Cy3 at the 5′ end. An appropriate amount of E. coli and Magnetospirillum magneticum AMB-1 cells was pretreated according to the same protocol used for preparing fixed MTB cells for FISH. They were then mixed with SHHR-1 cells and used as inner controls for both nonmagnetotactic Gammaproteobacteria and magnetotactic Alphaproteobacteria.

**TABLE 1:** FISH probes used in this study

Name	Target group	Target molecule	Sequence (5′ to 3′)	Positions	Reference or source
EUB338	Most bacteria	16S rRNA	GCTGCCTCCCGTAGGAGT	338–355	Amann et al. (26)
ALF968	Alphaproteobacteria	16S rRNA	GGTAAGGTTCTGCGCGTT	968–985	Neef (56)
GAM42a	Gammaproteobacteria	23S rRNA	GCCTTCCCACATCGTTT	1027–1043	Manz et al. (60)
SRB385Db	Desulfobacterales	16S rRNA	CGGCGTTGCTGCGTCAGG	385–402	Rabus et al. (58)
BaP	M. bavaricum, M. bavaricum-like MTB	16S rRNA	GCCATCCCCTCGCTTACT	655–672	Spring et al. (55); Lin et al. (49)
SHHR838	SHHR-1	16S rRNA	ACCCTTTTATGAGTCCAACGGCT	838–860	This study

FISH was carried out as described previously (72). Briefly, 1 μl of cell mixture (E. coli plus SHHR-1 or AMB-1 plus SHHR-1) was dropped directly on a high-precision cover glass (Paul Marienfeld GmbH & Co. KG, Germany), dried in air at ambient temperature, and gradually dehydrated in 50, 80, and 100% ethanol baths (3 min in each bath). To simplify the experiments and increase the comparability, all *in situ* hybridization experiments were performed at 46°C for 3 h in 9 μl of hybridization buffer (0.9 M NaCl, 20 mM Tris-HCl [pH 7.5], 0.02% [wt/vol] sodium dodecyl sulfate [SDS], 35% [vol/vol] formamide), which was mixed with 1 μl of EUB338 probe (50 ng/μl) and 1 μl of the probe of interest (50 ng/μl). The addition of 35% formamide to the hybridization buffer guarantees that the *T_m_* values of all the tested oligonucleotide probes can be at or slightly lower than 46°C (73). After 3 h of hybridization, samples were incubated in a washing buffer (0.08 M NaCl, 20 mM Tris-HCl [pH 7.5], 5 mM EDTA [pH 8.0], 0.01% [wt/vol] SDS) at 48°C for 30 min. Due to simultaneous *in situ* hybridization of two differently labeled fluorescent probes, a use of 30 min of washing time was performed as far as possible to wash out unspecific probe hybridizations. Samples were washed in Milli-Q water three times, dried in air at ambient temperature, and observed using an Olympus BX51 epifluorescence microscope.

For liquid-based FISH, about 100 μl of fixed cells (E. coli plus SHHR-1) was dehydrated in a graded ethanol series (50 to 100%) by centrifugation. After the removal of ethanol by centrifugation, the cell pellets were resuspended in 90 μl of hybridization buffer, 10 μl of EUB338 probe (50 ng/μl), and 10 μl of the probe of interest (50 ng/μl). They were then incubated for *in situ* hybridization at 46°C for 3 h. Afterwards, the samples were washed three times with washing buffer and then three times in Milli-Q water. Finally, washed cells were deposited onto letter-indexed carbon-coated TEM grids, dried in air at ambient temperature, and observed using the Olympus BX51 epifluorescence microscope.

After fluorescence microscopy analysis, cells mounted on cover glasses were coated with carbon using the Leica ACE200 low-vacuum sputter coater (Leica Microsystems, Wetzlar, Germany) and imaged using a Zeiss Ultra-55 field-emission scanning electron microscope (Carl Zeiss, Germany) operating at 5 kV. Cells mounted onto TEM grids were not sputter coated before the TEM observations. Generally, those areas/cells of interested which were at the sample edge on the cover glass and at the sample center on the TEM grid are easily coordinated by subsequent SEM and TEM determination, respectively, after fluorescence microscopy observations.

### STEM-HAADF, STEM-EDXS elemental mapping, and HRTEM analyses.

Magnetosome biomineralization was investigated using a JEM-2100F microscope (JEOL Ltd., Tokyo, Japan) operating at 200 kV equipped with a field emission gun, an ultrahigh-resolution (UHR) pole piece, and a Gatan energy filter (GIF2001) system (Gatan, Inc., Pleasanton, CA, USA). High-angle annular dark-field scanning transmission electron microscopy (HAADF-STEM) was used for Z-contrast imaging. Chemical compositional analysis was performed by energy-dispersive X-ray spectroscopy (EDXS) elemental mapping in the HAADF-STEM mode. Using this method, nanometer-scale spatial distribution of most elements can be determined, and the relative abundance of each element at a certain location can be semiquantitatively calculated (74). The length (along the long axis) and width (perpendicular to the long axis) of the magnetosomes were measured from TEM images. The shape factor was defined as width/length.

Magnetosome crystal habits were determined by the combination of Fourier analysis of HRTEM imaging of individual particles and crystallographic investigations, as previously described (38, 62). Briefly, for each particle, the zone axis and in-plane crystallographic directions were determined from the 2D fast Fourier transform (FFT) of the HRTEM image, allowing deduction of the corresponding stereographic projection. Idealized shapes that were modeled with the KrystalShaper software package (JCrystalSoft) were then compared to the observed shapes.

### Accession number(s).

The sequence of the 16S rRNA gene was deposited in GenBank under accession no. KX344069.

## Supplementary Material

Supplemental material

## References

[1] Uebe R, Schüler D 2016 Magnetosome biogenesis in magnetotactic bacteria. Nat Rev Microbiol 14:621–637. doi:10.1038/nrmicro.2016.99. 27620945

[2] Faivre D, Godec TU 2015 From bacteria to mollusks: the principles underlying the biomineralization of iron oxide materials. Angew Chem Int Ed Engl 54:4728–4747. doi:10.1002/anie.201408900. 25851816

[3] Pósfai M, Kasama T, Dunin-Borkowski RE 2013 Biominerals at the nanoscale: transmission electron microscopy methods for studying the special properties of biominerals. Eur Mineral Union Notes Mineral 14:377–435.

[4] Bazylinski DA, Frankel RB 2004 Magnetosome formation in prokaryotes. Nat Rev Microbiol 2:217–230. doi:10.1038/nrmicro842. 15083157

[5] Schüler D 2008 Genetics and cell biology of magnetosome formation in magnetotactic bacteria. FEMS Microbiol Rev 32:654–672. doi:10.1111/j.1574-6976.2008.00116.x. 18537832

[6] Komeili A 2012 Molecular mechanisms of compartmentalization and biomineralization in magnetotactic bacteria. FEMS Microbiol Rev 36:232–255. doi:10.1111/j.1574-6976.2011.00315.x. 22092030 PMC3540109

[7] Faivre D, Schüler D 2008 Magnetotactic bacteria and magnetosomes. Chem Rev 108:4875–4898. doi:10.1021/cr078258w. 18855486

[8] Pósfai M, Lefèvre C, Trubitsyn D, Bazylinski DA, Frankel RB 2013 Phylogenetic significance of composition and crystal morphology of magnetosome minerals. Front Microbiol 4:344. doi:10.3389/fmicb.2013.00344. 24324461 PMC3840360

[9] Li JH, Benzerara K, Bernard S, Beyssac O 2013 The link between biomineralization and fossilization of bacteria: Insights from field and experimental studies. Chem Geol 359:49–69. doi:10.1016/j.chemgeo.2013.09.013.

[10] Lefèvre CT, Bazylinski DA 2013 Ecology, diversity, and evolution of magnetotactic bacteria. Microbiol Mol Biol Rev 77:497–526. doi:10.1128/MMBR.00021-13. 24006473 PMC3811606

[11] Bellini S 2009 On a unique behavior of freshwater bacteria. Chin J Oceanol Limnol 27:3–5. doi:10.1007/s00343-009-0003-5.

[12] Alphandéry E, Chebbi I, Guyot F, Durand-Dubief M 2013 Use of bacterial magnetosomes in the magnetic hyperthermia treatment of tumours: a review. Int J Hyperthermia 29:801–809. doi:10.3109/02656736.2013.821527. 24024595

[13] Lefèvre CT, Bennet M, Landau L, Vach P, Pignol D, Bazylinski DA, Frankel RB, Klumpp S, Faivre D 2014 Diversity of magneto-aerotactic behaviors and oxygen sensing mechanisms in cultured magnetotactic bacteria. Biophys J 107:527–538. doi:10.1016/j.bpj.2014.05.043. 25028894 PMC4104051

[14] Frankel RB, Bazylinski DA, Johnson MS, Taylor BL 1997 Magneto-aerotaxis in marine coccoid bacteria. Biophys J 73:994–1000. doi:10.1016/S0006-3495(97)78132-3. 9251816 PMC1180996

[15] Roberts AP, Florindo F, Chang L, Heslop D, Jovane L, Larrasoaña JC 2013 Magnetic properties of pelagic marine carbonates. Earth-Sci Rev 127:111–139. doi:10.1016/j.earscirev.2013.09.009.

[16] Kopp RE, Kirschvink JL 2008 The identification and biogeochemical interpretation of fossil magnetotactic bacteria. Earth-Sci Rev 86:42–61. doi:10.1016/j.earscirev.2007.08.001.

[17] Liu SZ, Deng CL, Xiao JL, Li JH, Paterson GA, Chang L, Yi L, Qin HF, Pan YX, Zhu RX 2015 Insolation driven biomagnetic response to the Holocene Warm Period in semi-arid East Asia. Sci Rep 5:8001. doi:10.1038/srep08001. 25614046 PMC4303925

[18] Pan YX, Petersen N, Davila AF, Zhang LM, Winklhofer M, Liu QS, Hanzlik M, Zhu RX 2005 The detection of bacterial magnetite in recent sediments of Lake Chiemsee (southern Germany). Earth Planet Sci Lett 232:109–123. doi:10.1016/j.epsl.2005.01.006.

[19] Staniland S, Williams W, Telling N, Van Der Laan G, Harrison A, Ward B 2008 Controlled cobalt doping of magnetosomes *in vivo*. Nat Nanotechnol 3:158–162. doi:10.1038/nnano.2008.35. 18654488

[20] Li JH, Menguy N, Arrio M-A, Sainctavit P, Juhin A, Wang YZ, Chen HT, Bunau O, Otero E, Ohresser P, Pan YX 2016 Controlled cobalt doping in the spinel structure of magnetosome magnetite: new evidences from element- and site-specific X-ray magnetic circular dichroism analyses. J R Soc Interface 13:20160355. doi:10.1098/rsif.2016.0355. 27512138 PMC5014062

[21] Kolinko S, Richter M, Glöckner F-O, Brachmann A, Schüler D 2016 Single-cell genomics of uncultivated deep-branching magnetotactic bacteria reveals a conserved set of magnetosome genes. Environ Microbiol 18:21–37. doi:10.1111/1462-2920.12907. 26060021

[22] Kolinko S, Jogler C, Katzmann E, Wanner G, Peplies J, Schüler D 2012 Single-cell analysis reveals a novel uncultivated magnetotactic bacterium within the candidate division OP3. Environ Microbiol 14:1709–1721. doi:10.1111/j.1462-2920.2011.02609.x. 22003954

[23] Wagner M, Haider S 2012 New trends in fluorescence *in situ* hybridization for identification and functional analyses of microbes. Curr Opin Biotechnol 23:96–102. doi:10.1016/j.copbio.2011.10.010. 22079351

[24] Amann R, Fuchs BM 2008 Single-cell identification in microbial communities by improved fluorescence *in situ* hybridization techniques. Nat Rev Microbiol 6:339–348. doi:10.1038/nrmicro1888. 18414500

[25] Wagner M, Horn M, Daims H 2003 Fluorescence *in situ* hybridisation for the identification and characterisation of prokaryotes. Curr Opin Microbiol 6:302–309. doi:10.1016/S1369-5274(03)00054-7. 12831908

[26] Amann RI, Krumholz L, Stahl DA 1990 Fluorescent-oligonucleotide probing of whole cells for determinative, phylogenetic, and environmental studies in microbiology. J Bacteriol 172:762–770. doi:10.1128/jb.172.2.762-770.1990. 1688842 PMC208504

[27] DeLong EF, Wickham GS, Pace NR 1989 Phylogenetic stains: ribosomal RNA-based probes for the identification of single cells. Science 243:1360–1363. doi:10.1126/science.2466341. 2466341

[28] Taoka A, Kondo J, Oestreicher Z, Fukumori Y 2014 Characterization of uncultured giant rod-shaped magnetotactic *Gammaproteobacteria* from a fresh water pond in Kanazawa, Japan. Microbiology 160:2226–2234. doi:10.1099/mic.0.078717-0. 25028459

[29] Wang YZ, Lin W, Li JH, Pan YX 2013 High diversity of magnetotactic deltaproteobacteria in a freshwater niche. Appl Environ Microbiol 79:2813–2817. doi:10.1128/AEM.03635-12. 23377941 PMC3623179

[30] Lin W, Li JH, Pan YX 2012 Newly isolated but uncultivated magnetotactic bacterium of the phylum *Nitrospirae* from Beijing, China. Appl Environ Microbiol 78:668–675. doi:10.1128/AEM.06764-11. 22113917 PMC3264100

[31] Lin W, Jogler C, Schüler D, Pan Y 2011 Metagenomic analysis reveals unexpected subgenomic diversity of magnetotactic bacteria within the *Nitrospirae phylum*. Appl Environ Microbiol 77:323–326. doi:10.1128/AEM.01476-10. 21057016 PMC3019724

[32] Pan HM, Zhu KL, Song T, Yu-Zhang K, Lefevre C, Xing S, Liu M, Zhao SJ, Xiao T, Wu LF 2008 Characterization of a homogeneous taxonomic group of marine magnetotactic cocci within a low tide zone in the China Sea. Environ Microbiol 10:1158–1164. doi:10.1111/j.1462-2920.2007.01532.x. 18279350

[33] Flies CB, Peplies J, Schüler D 2005 Combined approach for characterization of uncultivated magnetotactic bacteria from various aquatic environments. Appl Environ Microbiol 71:2723–2731. doi:10.1128/AEM.71.5.2723-2731.2005. 15870364 PMC1087566

[34] Spring S, Amann R, Ludwig W, Schleifer KH, Schüler D, Poralla K, Petersen N 1995 Phylogenetic analysis of uncultured magnetotactic bacteria from the alpha-subclass of *Proteobacteria*. Syst Appl Microbiol 17:501–508. doi:10.1016/S0723-2020(11)80068-8.

[35] Spring S, Amann R, Ludwig W, Schleifer KH, Petersen N 1992 Phylogenetic diversity and identification of nonculturable magnetotactic bacteria. Syst Appl Microbiol 15:116–122. doi:10.1016/S0723-2020(11)80147-5.

[36] Brydson R, Brown A, Benning LG, Livi K 2014 Analytical transmission electron microscopy, p 219–269. *In* Henderson GS, Neuville DR, Downs RT (ed), Spectroscopic methods in mineralology and materials sciences, vol 78 Mineralogical Society of America, Chantilly, VA.

[37] Li JH, Pan YX 2015 Applications of transmission electron microscopy in the earth sciences. Scientia Sinica Terrae 45:1359–1382. (In Chinese.) doi:10.1360/zd2015-45-09-1359.

[38] Li JH, Menguy N, Gatel C, Boureau V, Snoeck E, Patriarche G, Leroy E, Pan YX 2015 Crystal growth of bullet-shaped magnetite in magnetotactic bacteria of the *Nitrospirae phylum*. J R Soc Interface 12:20141288. doi:10.1098/rsif.2014.1288. 25566884 PMC4305428

[39] Endesfelder U 2015 Advances in correlative single-molecule localization microscopy and electron microscopy. NanoBioImaging 1:29–37. doi:10.2478/nbi-2014-0002.

[40] Giepmans BNG 2008 Bridging fluorescence microscopy and electron microscopy. Histochem Cell Biol 130:211–217. doi:10.1007/s00418-008-0460-5. 18575880 PMC2491700

[41] Dukes MJ, Peckys DB, de Jonge N 2010 Correlative fluorescence microscopy and scanning transmission electron microscopy of quantum dot labeled proteins in whole cells in liquid. ACS Nano 4:4110–4116. doi:10.1021/nn1010232. 20550177 PMC2919632

[42] Schmidt H, Eickhorst T, Mußmann M 2012 Gold-FISH: A new approach for the *in situ* detection of single microbial cells combining fluorescence and scanning electron microscopy. Syst Appl Microbiol 35:518–525. doi:10.1016/j.syapm.2012.04.006. 22770611

[43] Spring S, Lins U, Amann R, Schleifer K, Ferreira L, Esquivel D, Farina M 1998 Phylogenetic affiliation and ultrastructure of uncultured magnetic bacteria with unusually large magnetosomes. Arch Microbiol 169:136–147. doi:10.1007/s002030050553. 9446685

[44] Knierim B, Luef B, Wilmes P, Webb RI, Auer M, Comolli LR, Banfield JF 2012 Correlative microscopy for phylogenetic and ultrastructural characterization of microbial communities. Environ Microbiol Rep 4:36–41. doi:10.1111/j.1758-2229.2011.00275.x. 23757227 PMC4444221

[45] Lefèvre CT, Viloria N, Schmidt ML, Posfai M, Frankel RB, Bazylinski DA 2012 Novel magnetite-producing magnetotactic bacteria belonging to the *Gammaproteobacteria*. ISME J 6:440–450. doi:10.1038/ismej.2011.97. 21776027 PMC3260515

[46] Isambert A, Menguy N, Larquet E, Guyot F, Valet JP 2007 Transmission electron microscopy study of magnetites in a freshwater population of magnetotactic bacteria. Am Mineral 92:621–630. doi:10.2138/am.2007.2278.

[47] Devouard B, Pósfai M, Hua X, Bazylinski DA, Frankel RB, Buseck PR 1998 Magnetite from magnetotactic bacteria: size distributions and twinning. Am Mineral 83:1387–1398. doi:10.2138/am-1998-11-1228.

[48] Li JH, Pan YX, Chen GJ, Liu QS, Tian LX, Lin W 2009 Magnetite magnetosome and fragmental chain formation of *Magnetospirillum magneticum* AMB-1: transmission electron microscopy and magnetic observations. Geophys J Int 177:33–42. doi:10.1111/j.1365-246X.2009.04043.x.

[49] Lin W, Li JH, Schüler D, Jogler C, Pan YX 2009 Diversity analysis of magnetotactic bacteria in Lake Miyun, northern China, by restriction fragment length polymorphism. Syst Appl Microbiol 32:342–350. doi:10.1016/j.syapm.2008.10.005. 19168303

[50] Kanemaru T, Hirata K, Takasu S, Isobe S, Mizuki K, Mataka S, Nakamura K-i 2009 A fluorescence scanning electron microscope. Ultramicroscopy 109:344–349. doi:10.1016/j.ultramic.2009.01.002. 19211187

[51] Woehl TJ, Kashyap S, Firlar E, Perez-Gonzalez T, Faivre D, Trubitsyn D, Bazylinski DA, Prozorov T 2014 Correlative electron and fluorescence microscopy of magnetotactic bacteria in liquid: toward *in vivo* imaging. Sci Rep 4:6854. doi:10.1038/srep06854. 25358460 PMC4215306

[52] Huang WE, Stoecker K, Griffiths R, Newbold L, Daims H, Whiteley AS, Wagner M 2007 Raman-FISH: combining stable-isotope Raman spectroscopy and fluorescence *in situ* hybridization for the single cell analysis of identity and function. Environ Microbiol 9:1878–1889. doi:10.1111/j.1462-2920.2007.01352.x. 17635536

[53] Behrens S, Losekann T, Pett-Ridge J, Weber PK, Ng WO, Stevenson BS, Hutcheon ID, Relman DA, Spormann AM 2008 Linking microbial phylogeny to metabolic activity at the single-cell level by using enhanced element labeling-catalyzed reporter deposition fluorescence *in situ* hybridization (EL-FISH) and NanoSIMS. Appl Environ Microbiol 74:3143–3150. doi:10.1128/AEM.00191-08. 18359832 PMC2394947

[54] Cosmidis J, Benzerara K 2014 Soft X-ray scanning transmission micro-spectroscopy. *In* Gower L, DiMasi E (ed), Handbook of biomineralization. Taylor and Francis, London, United Kingdom.

[55] Spring S, Amann R, Ludwig W, Schleifer KH, van Gemerden H, Petersen N 1993 Dominating role of an unusual magnetotactic bacterium in the microaerobic zone of a freshwater sediment. Appl Environ Microbiol 59:2397–2403. 16349008 10.1128/aem.59.8.2397-2403.1993 PMC182297

[56] Neef A 1997 Anwendung der in situ-Einzelzellidentifizierung von Bakterien zur Populationsanalyse in komplexen mikrobiellen Biozönosen. Ph.D. thesis Technical University Munich, Munich, Germany.

[57] Lin W, Pan YX 2009 Specific primers for the detection of freshwater Ga magnetotactic cocci. Int Microbiol 12:237–242. 20112228

[58] Rabus R, Fukui M, Wilkes H, Widdle F 1996 Degradative capacities and 16S rRNA-targeted whole-cell hybridization of sulfate-reducing bacteria in an anaerobic enrichment culture utilizing alkylbenzenes from crude oil. Appl Environ Microbiol 62:3605–3613. 8837415 10.1128/aem.62.10.3605-3613.1996 PMC168167

[59] Sakaguchi T, Arakaki A, Matsunaga T 2002 *Desulfovibrio magneticus* sp. nov., a novel sulfate-reducing bacterium that produces intracellular single-domain-sized magnetite particles. Int J Syst Evol Microbiol 52:215–221. doi:10.1099/00207713-52-1-215. 11837306

[60] Manz W, Amann R, Ludwig W, Wagner M, Schleifer K-H 1992 Phylogenetic oligodeoxynucleotide probes for the major subclasses of proteobacteria: problems and solutions. Syst Appl Microbiol 15:593–600. doi:10.1016/S0723-2020(11)80121-9.

[61] Leão P, Teixeira LCRS, Cypriano J, Farina M, Abreu F, Bazylinski DA, Lins U 2016 North-seeking magnetotactic *Gammaproteobacteria* in the Southern Hemisphere. Appl Environ Microbiol 82:5595–5602. doi:10.1128/AEM.01545-16. 27401974 PMC5007775

[62] Faivre D, Menguy N, Pósfai M, Schüler D 2008 Environmental parameters affect the physical properties of fast-growing magnetosomes. Am Mineral 93:463–469. doi:10.2138/am.2008.2678.

[63] Buseck PR, Dunin-Borkowski RE, Devouard B, Frankel RB, McCartney MR, Midgley PA, Pósfai M, Weyland M 2001 Magnetite morphology and life on Mars. Proc Natl Acad Sci U S A 98:13490–13495. doi:10.1073/pnas.241387898. 11717421 PMC61068

[64] Schüler D 2002 The biomineralization of magnetosomes in *Magnetospirillum gryphiswaldense*. Int Microbiol 5:209–214. doi:10.1007/s10123-002-0086-8. 12497187

[65] Li JH, Pan YX, Liu QS, Yu-Zhang K, Menguy N, Che RC, Qin HF, Lin W, Wu WF, Petersen N, Yang XA 2010 Biomineralization, crystallography and magnetic properties of bullet-shaped magnetite magnetosomes in giant rod magnetotactic bacteria. Earth Planet Sci Lett 293:368–376. doi:10.1016/j.epsl.2010.03.007.

[66] Lane DJ 1991 16S/23S rRNA sequencing. *In* Stackebrandt E, Goodfellow M (ed), Nucleic acid techniques in bacterial systematics. John Wiley & Sons, Chichester, United Kingdom.

[67] Chenna R, Sugawara H, Koike T, Lopez R, Gibson TJ, Higgins DG, Thompson JD 2003 Multiple sequence alignment with the Clustal series of programs. Nucleic Acids Res 31:3497–3500. doi:10.1093/nar/gkg500. 12824352 PMC168907

[68] Kumar S, Stecher G, Tamura K 2016 MEGA7: Molecular Evolutionary Genetics Analysis version 7.0 for bigger datasets. Mol Biol Evol 33:1870–1874. doi:10.1093/molbev/msw054. 27004904 PMC8210823

[69] Greuter D, Loy A, Horn M, Rattei T 2016 ProbeBase—an online resource for rRNA-targeted oligonucleotide probes and primers: new features 2016. Nucleic Acids Res 44:D586–D589. doi:10.1093/nar/gkv1232. 26586809 PMC4702872

[70] Loy A, Arnold R, Tischler P, Rattei T, Wagner M, Horn M 2008 ProbeCheck—a central resource for evaluating oligonucleotide probe coverage and specificity. Environ Microbiol 10:2894–2898. doi:10.1111/j.1462-2920.2008.01706.x. 18647333 PMC2613240

[71] Kibbe WA 2007 OligoCalc: an online oligonucleotide properties calculator. Nucleic Acids Res 35:W43–W46. doi:10.1093/nar/gkm234. 17452344 PMC1933198

[72] Pernthaler A, Pernthaler J, Amann R 2002 Fluorescence *in situ* hybridization and catalyzed reporter deposition for the identification of marine bacteria. Appl Environ Microbiol 68:3094–3101. doi:10.1128/AEM.68.6.3094-3101.2002. 12039771 PMC123953

[73] Pernthaler J, Glöckner FO, Schönhuber W, Amann R 2001 Fluorescence *in situ* hybridization with rRNA-targeted oligonucleotide probes. *In* Paul J (ed), Methods in microbiology: marine microbiology, vol 30 Academic Press Ltd., London, United Kingdom.

[74] Li JH, Margaret Oliver I, Cam N, Boudier T, Blondeau M, Leroy E, Cosmidis J, Skouri-Panet F, Guigner J-M, Férard C, Poinsot M, Moreira D, Lopez-Garcia P, Cassier-Chauvat C, Chauvat F, Benzerara K 2016 Biomineralization patterns of intracellular carbonatogenesis in cyanobacteria: molecular hypotheses. Minerals 6:10. doi:10.3390/min6010010.

