# Supplementary material for: Single-Cell Resolution of Uncultured Magnetotactic Bacteria via Fluorescence-Coupled Electron Microscopy
Source: Appl Environ Microbiol. 2017 May 31;83(12):e00409-17. doi: 10.1128/AEM.00409-17 (PMC5452806; doi:10.1128/AEM.00409-17)
Supplement: Supplemental material [file supp_83_12_e00409-17__index.html]

Supplemental material 

# Single-Cell Resolution of Uncultured Magnetotactic Bacteria via Fluorescence-Coupled Electron Microscopy

## Supplemental material

- Supplemental file 1 -

  FISH identification (Fig. S1), FISH-SEM (Fig. S2), and FISH-TEM (Fig. S3) of SHHR-1 cells, morphological features of SHHR-1 cells and their magnetosomes (Fig. S4), STEM-EDXS mapping analysis (Fig. S5), coupled FISH-SEM identification of uncultured MTB (Fig. S6 to S9), and HRTEM images and morphology modeling of SHHR-1 magnetosomes.

  PDF, 3.4M
