# Supplementary material for: Single-Cell Resolution of Uncultured Magnetotactic Bacteria via Fluorescence-Coupled Electron Microscopy
Source: Appl Environ Microbiol. 2017 May 31;83(12):e00409-17. doi: 10.1128/AEM.00409-17 (PMC5452806; doi:10.1128/AEM.00409-17)
Supplement: Supplemental material [file AEM.00409-17_zam999117880s1.pdf]

Supplementary Material-1

Supplementary Figures

Single-cell resolution study of uncultured magnetotactic bacteria via fluorescence-coupled electron microscopy

Jinhua Li, Heng Zhang, Nicolas Menguy, Karim Benzerara, Fuxian Wang, Xiaoting Lin, Zhibao Chen, Yongxin Pan

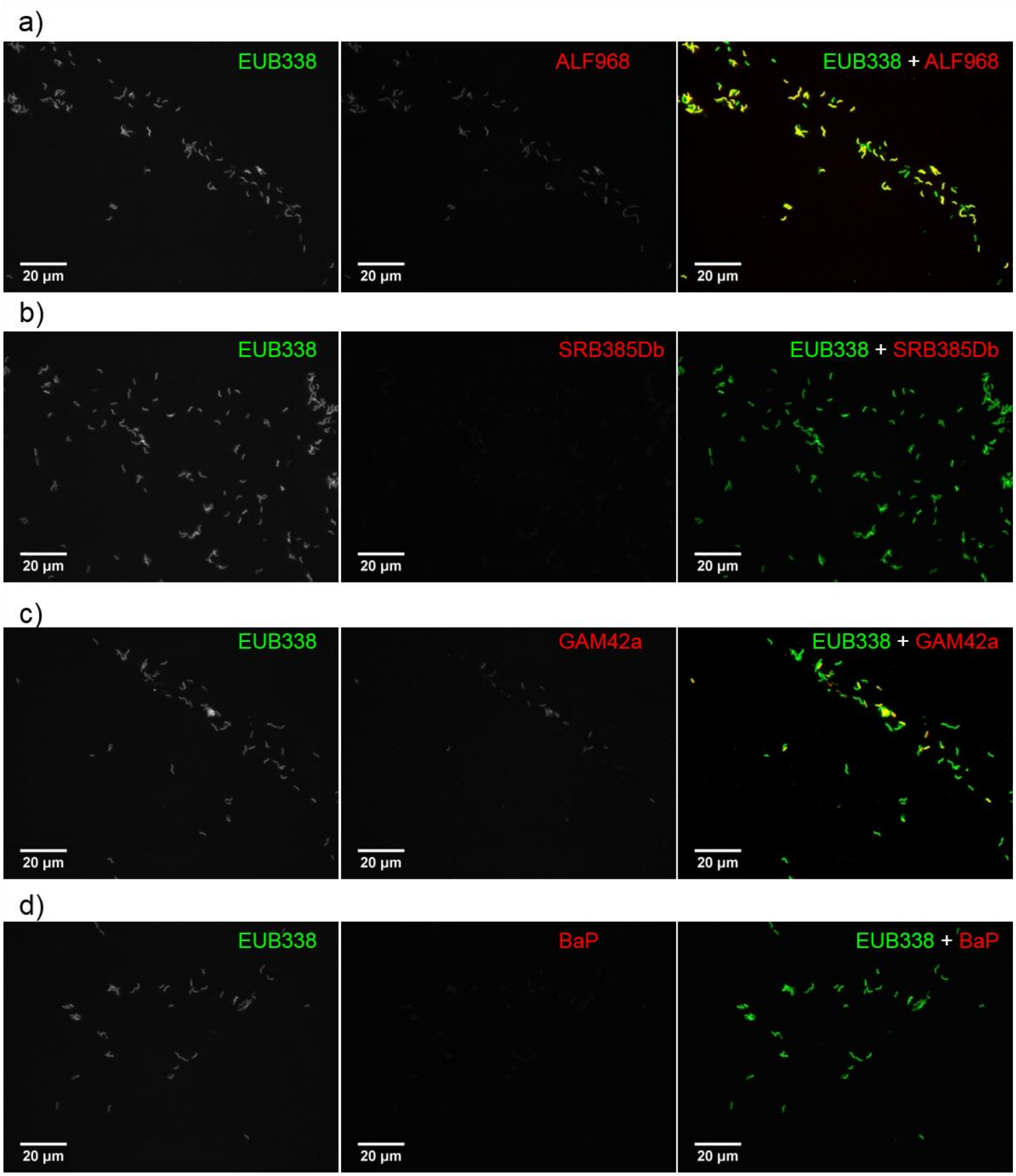

FIG S1 FISH identification of SHHR-1 cells with the *Alphaproteobacteria*-specific probe ALF968 (a), *Desulfobacteriaceae*-specific probe SRB385Db (b), *Gammaproteobacteria*-specific probe GAM42a (c), and '*Candidatus* Magnetobacterium bavaricum'-specific probe BaP (d). For each identification, the same microscopic field is shown followed by *in situ* hybridization with the 5'-FAM-labeled universal bacterial probe EUB338 (green) (the first column) and the group-specific probes (red) (the second column), and their overlapped fluorescence microscopy image (the third column). As expected, inner control AMB-1 cells were targeted both by the EUB338 and ALF968 probes, but not targeted by three other group-specific probes. While, the SHHR-1 cells were targeted both by the EUB338 and GAM42a probes, but not targeted by three other group-probes.

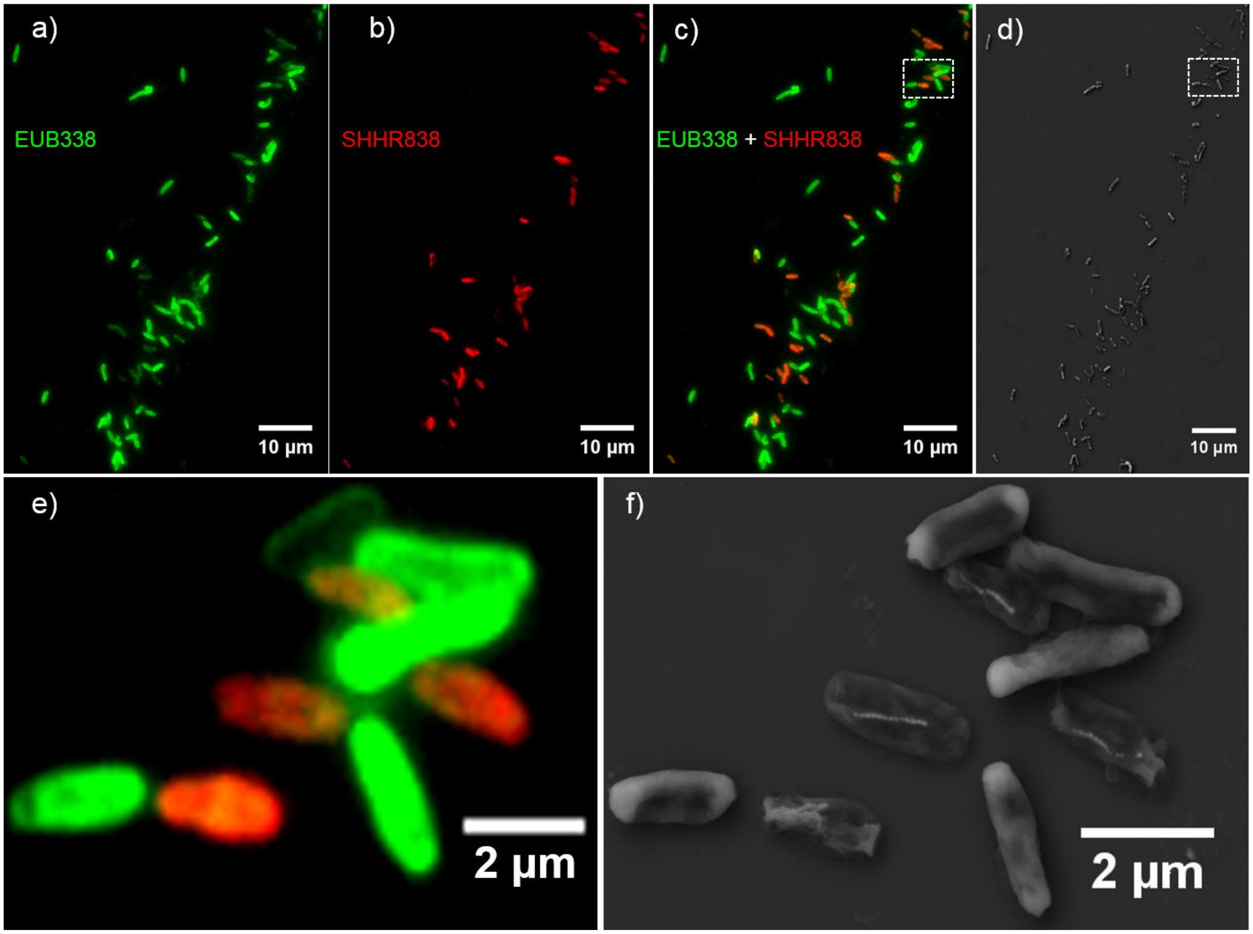

FIG S2 Coupled FISH-SEM identification of SHHR-1 cells. (a)-(c) Fluorescence microscopy images of SHHR-1 cells hybridized with the 5'-FAM-labeled universal bacterial probe EUB338 (green) and the 5'-Cy3-labeled SHHR-1-specific probe SHHR838 (red). SHHR-1 cells and inner control *E. coli* cells were fluorescently labeled with the EUB338 probe, as indicated by their green colors (a). Only some rod-shaped bacteria were fluorescently labeled with the SHHR838 probe, which is indicated by their red colors in image b, and by their yellow-red colors in the overlapped fluorescence microscopy image c. (d) Low-magnification SEM image of the same microscopy field as in c. (e) Close-up of the area indicated in image c by a white dashed square. (f) Large-magnification SEM image of the same field as in image e.

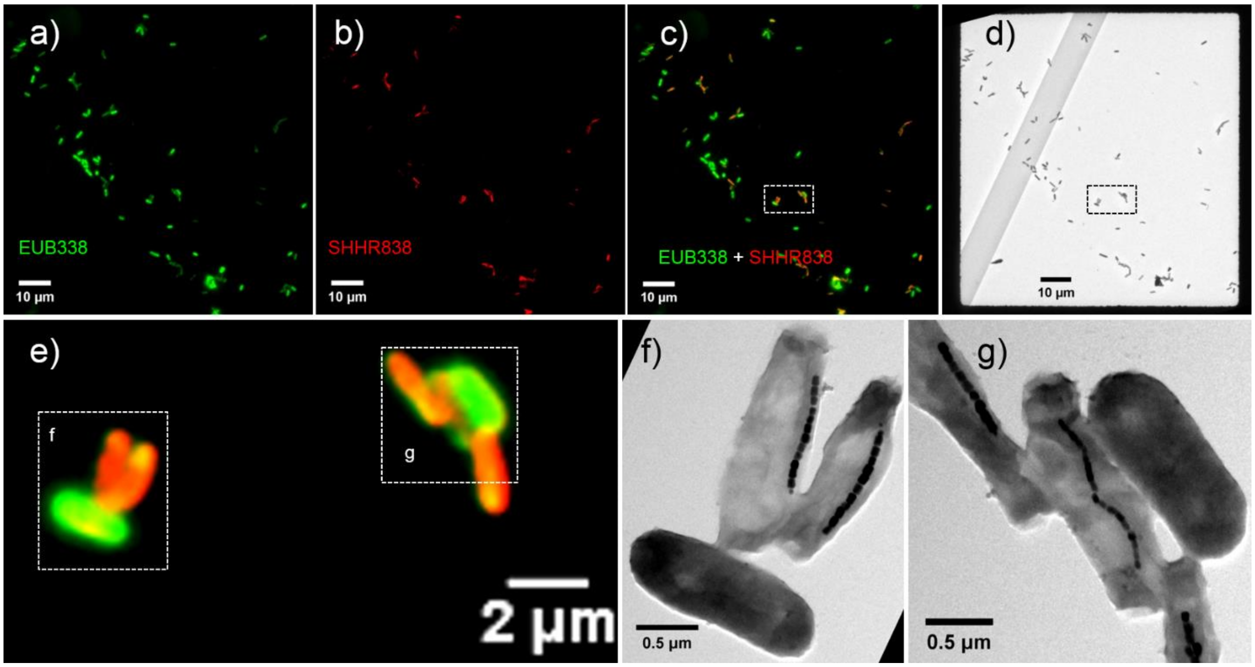

FIG S3 Coupled FISH-TEM identification of SHHR-1 cells. (a)-(c) Fluorescence microscopy image of SHHR-1 cells hybridized with the 5'-FAM-labeled universal bacterial probe EUB338 (green) and the 5'-Cy3-labeled SHHR-1-specific probe SHHR838 (red). Both SHHR-1 cells and inner control *E. coli* cells were fluorescently labeled with the EUB338 probe as shown by their green colors (a), while only some rod-shaped bacteria were fluorescently labeled with the SHHR838 probe as shown by their red colors in image b, and by their yellow-red colors in the overlapped fluorescence microscopy image c. (d) Low-magnification TEM image of the same field as in image c. (e) Close-up of the area indicated in image c by a white dashed square. (f) and (g) Large-magnification TEM images of the same microscopic area indicated in image e by a white dashed square and in image d by a black dashed square.

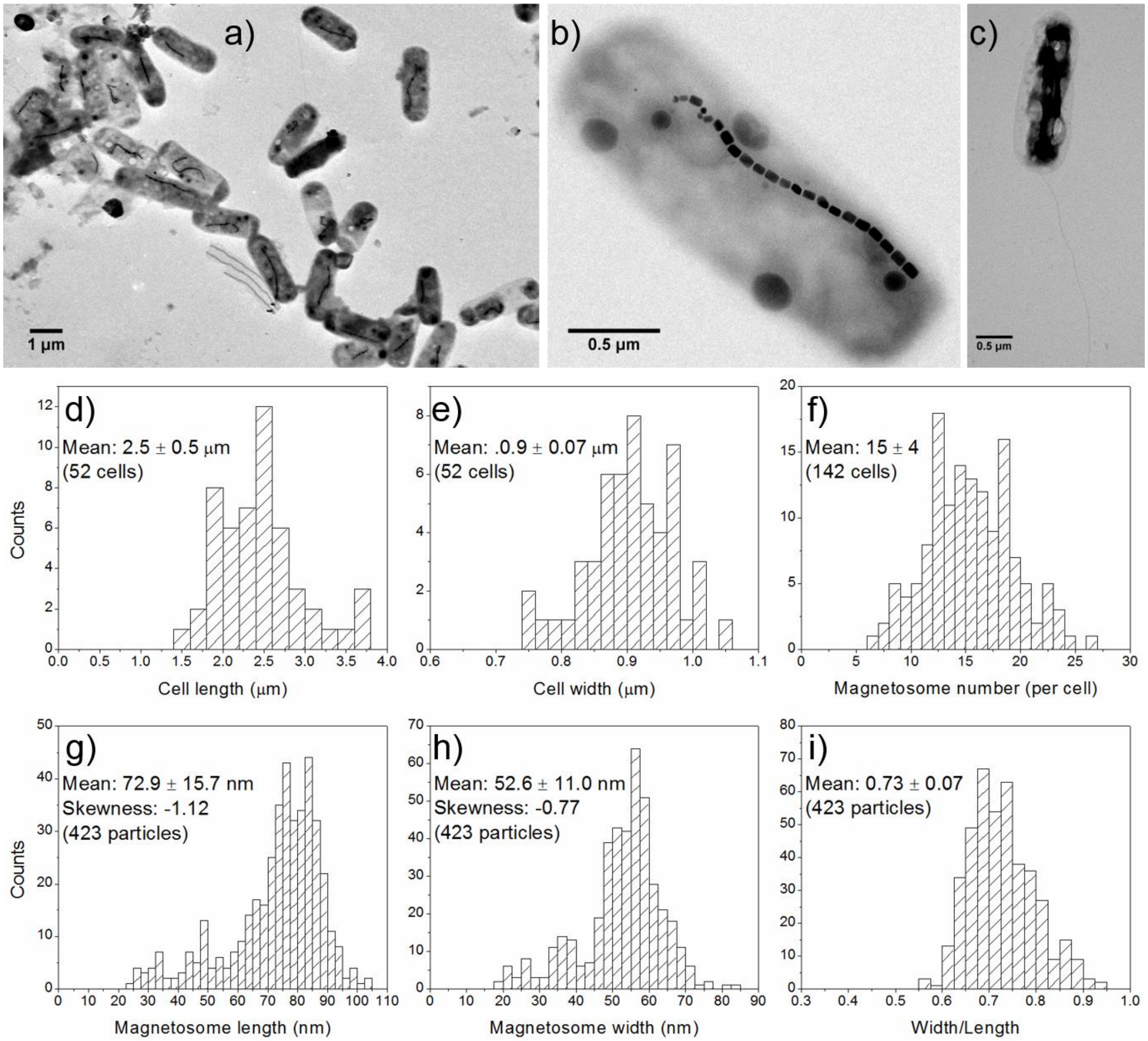

FIG S4 Morphological features of SHHR-1 cells and their magnetosomes. (a)-(c) Bright-field TEM images of many SHHR-1 cells (a), one SHHR-1 cell (b), and one negatively stained (1% uranyl acetate) SHHR-1 cell (c). (d)-(i) Histograms of cell length (d), cell width (e), magnetosome numbers per cell (f), magnetosome length (g), magnetosome width (h), and shape factor of magnetosomes (Width/Length) (i).

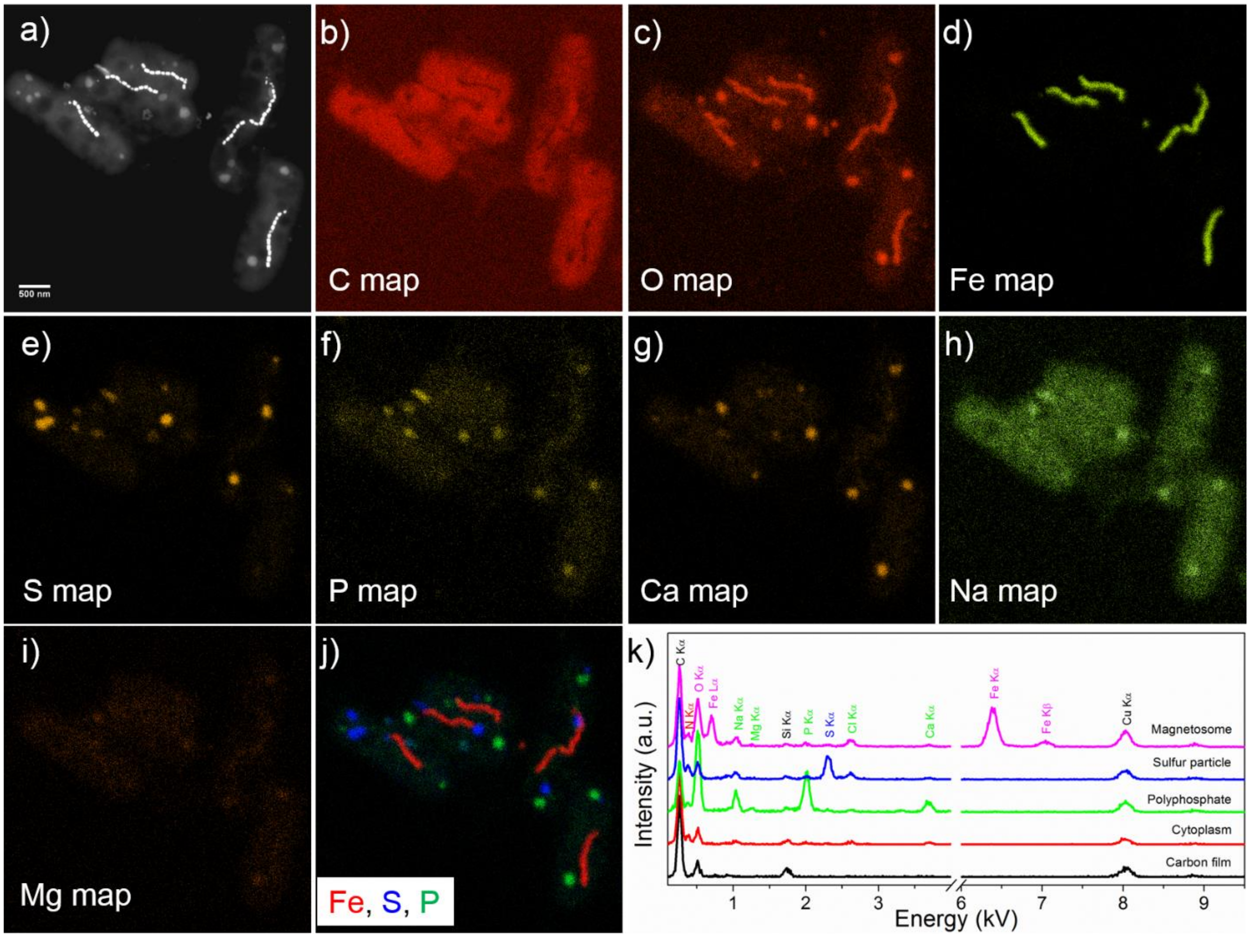

FIG S5. STEM-EDXS mapping (in HAADF mode) analysis of SHHR-1 cells. (a)-(i) HAADF-STEM image of SHHR-1 cells (a), and the corresponding chemical maps of C (C Kα) (b), O (O Kα) (c), Fe (Fe Kα) (d), S (S Kα) (e), P (P Kα) (f), Ca (Ca Kα) (g), Na (Na Kα) (h), and Mg (Mg Kα) (i). (j) Overlapped map of Fe (red), S (blue) and P (green). (k) EDX spectra extracted from pure carbon film of TEM grid (black line), cell cytoplasm (red line), polyphosphate granule (green line), sulfur-rich particle (blue line), and magnetosomes (pink line).

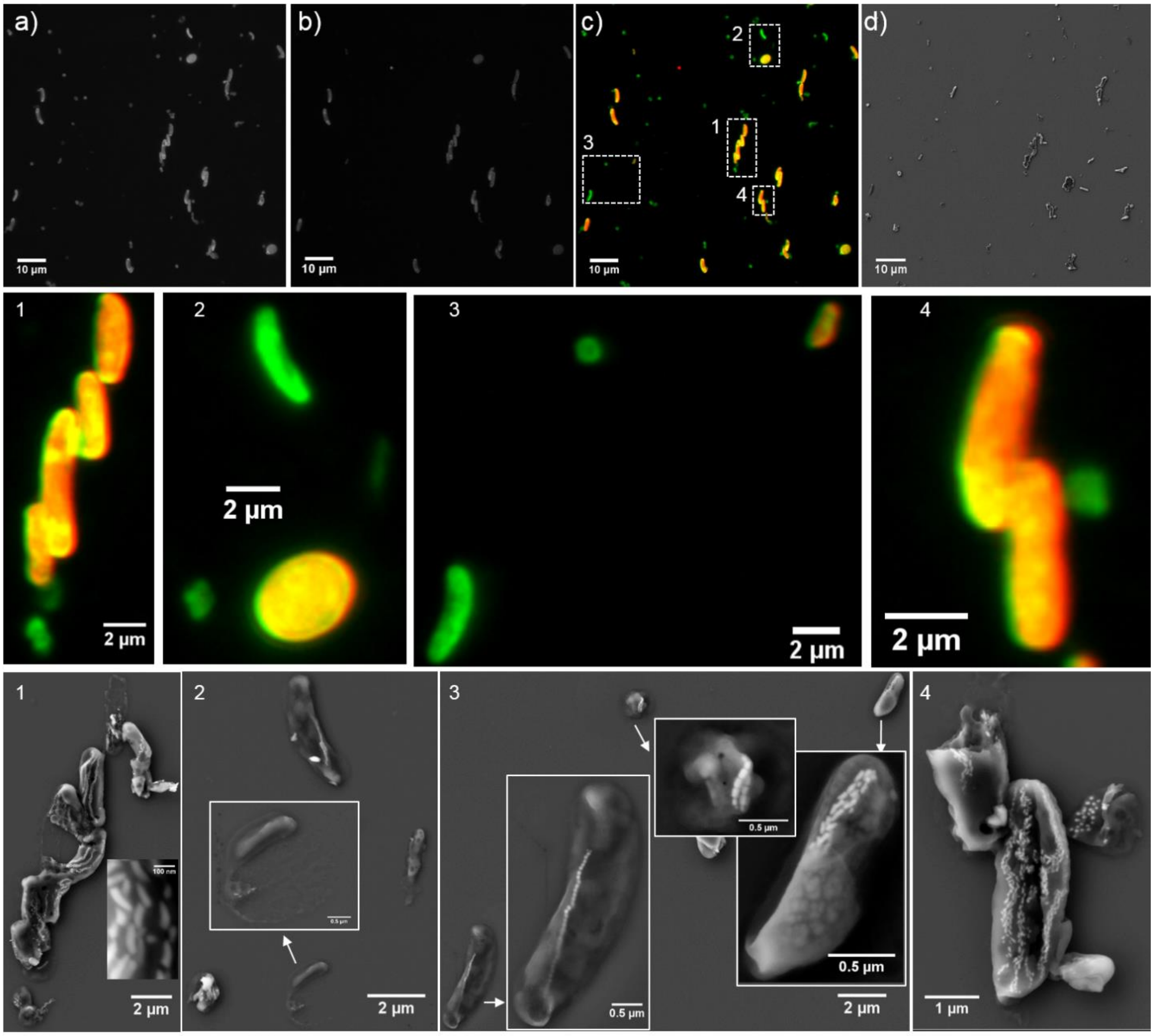

FIG S6 Coupled FISH-SEM identification of uncultured MTB in freshwater sediments collected from Lake Miyun in north Beijing in China based on *in situ* hybridization with the '*Candidatus* Magnetobacterium bavaricum'-specific probe BaP. The same microscopic field is shown following by *in situ* hybridization with the 5'-FAM-labeled universal bacterial probe EUB338 (green) (a) and the 5'-Cy3-labeled *Ca*. M. bavaricum-specific probe BaP (red) (b), and their overlapped fluorescence microscopy image (c). (d) Low-magnification SEM image of the same microscopic field of image c. The second lines show close-ups of fluorescence microscopy images indicated in image c by white dashed squares and numbers. The third lines show high-magnification SEM images of the same microscopic areas to the second line. The BaP probe can specifically target three kinds of magnetotactic *Nitrospirae*: large rod-shaped MYR-1, large watermelon-shaped MWB-1, and small rod-shaped MYR-2. All these three kinds of bacteria form curved, bullet-shaped magnetosomes arranged into bundle chains. Notably, some MYR-1 and MWB-1 cells have been partially destroyed due to vacuum treatment during carbon coating and before SEM observations.

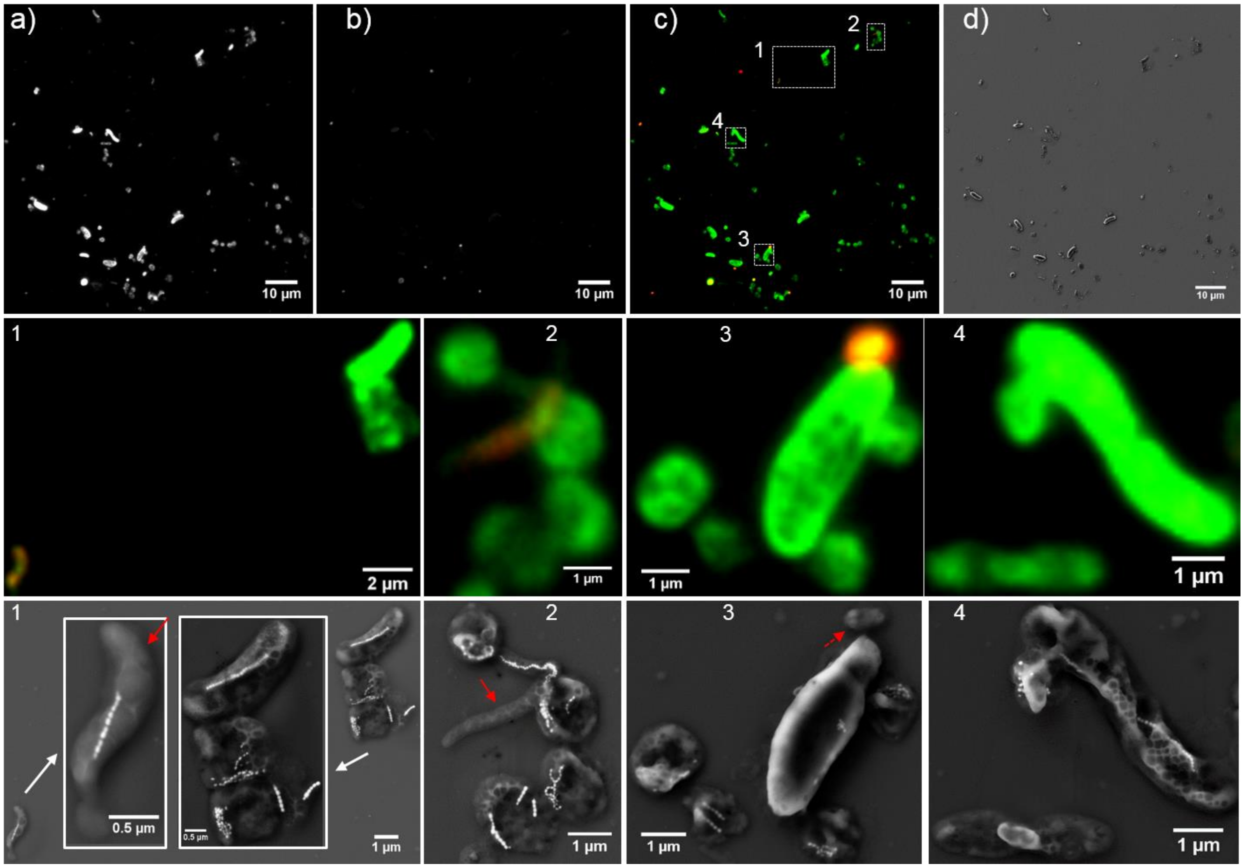

FIG S7 Coupled FISH-SEM identification of uncultured MTB in freshwater sediments collected from Lake Miyun in north Beijing in China based on *in situ* hybridization with the *Alphaproteobacteria*-specific probe ALF968. The same microscopic field is shown following by *in situ* hybridization with the 5'-FAM-labeled universal bacterial probe EUB338 (green) (a) and the 5'-Cy3-labeled *Alphaproteobacteria*-specific probe ALF968 (red) (b), and their overlapped fluorescence microscopy image (c). (d) Low-magnification SEM image of the same microscopic field of image c. The second lines show close-ups of fluorescence microscopy images indicated in image c by white dashed squares and numbers. The third lines show high-magnification SEM images of the same microscopic areas to the second line. Only spiral MTB cells (indicated by red solid lines with arrow heads) and non-magnetotactic bacteria cell (indicated by red dashed lines with arrow head) were targeted by the ALF968 probe.

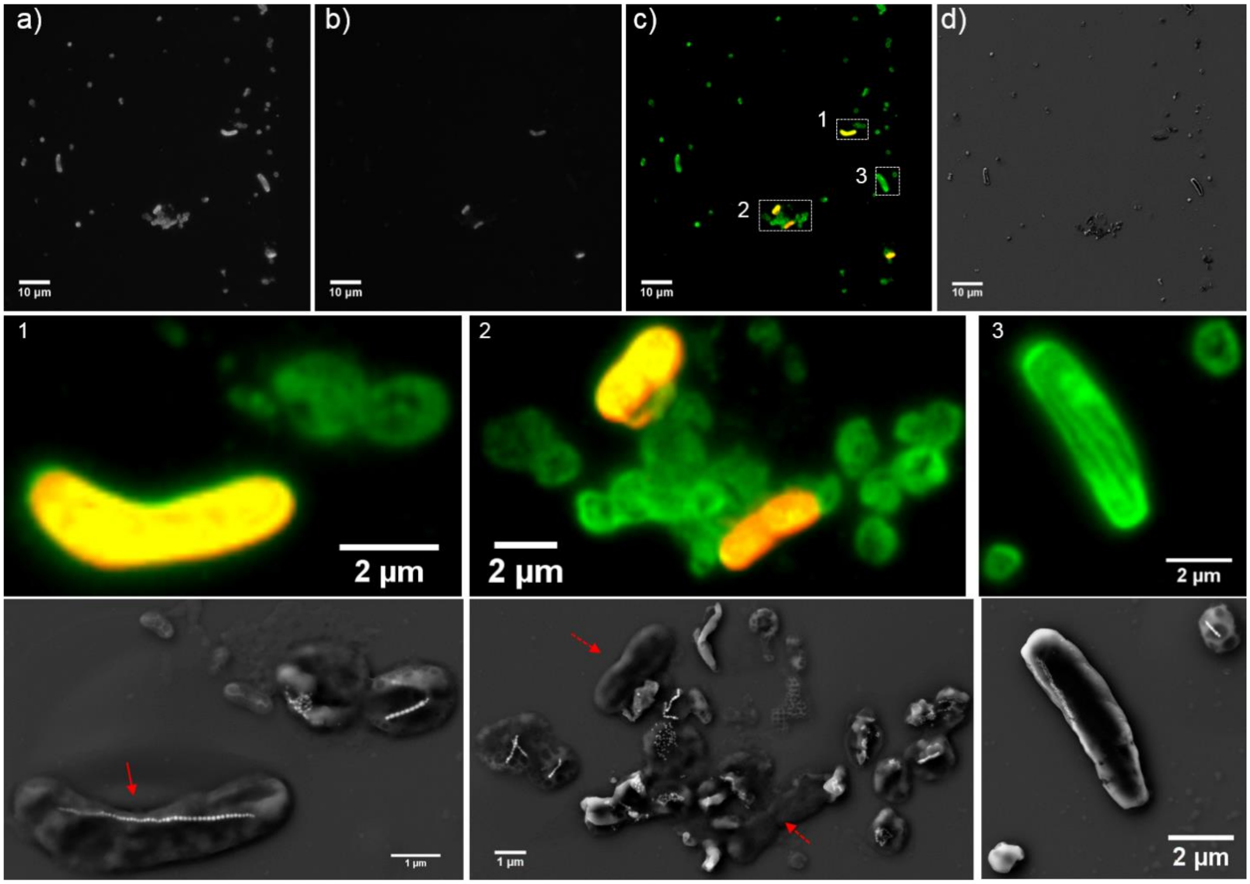

FIG S8 Coupled FISH-SEM identification of uncultured MTB in freshwater sediments collected from Lake Miyun in north Beijing in China based on *in situ* hybridization with the *Gammaproteobacteria*-specific probe GAM42a. The same microscopic field is shown following by *in situ* hybridization with the 5'-FAM-labeled universal bacterial probe EUB338 (green) (a) and the 5'-Cy3-labeled *Gammaproteobacteria*-specific probe GAM42a (red) (b), and their overlapped fluorescence microscopy image (c). (d) Low-magnification SEM image of the same microscopic field of image c. The second lines show close-ups of fluorescence microscopy images indicated in image c by white dashed squares and numbers. The third lines show high-magnification SEM images of the same microscopic areas to the second line. One giant rod-shaped MTB (tentatively named as MYR-3) (indicated by red solid line with arrowhead) and some non-magnetotactic bacteria (indicated by red dashed lines with arrow heads) were targeted by the GAM42a probe.

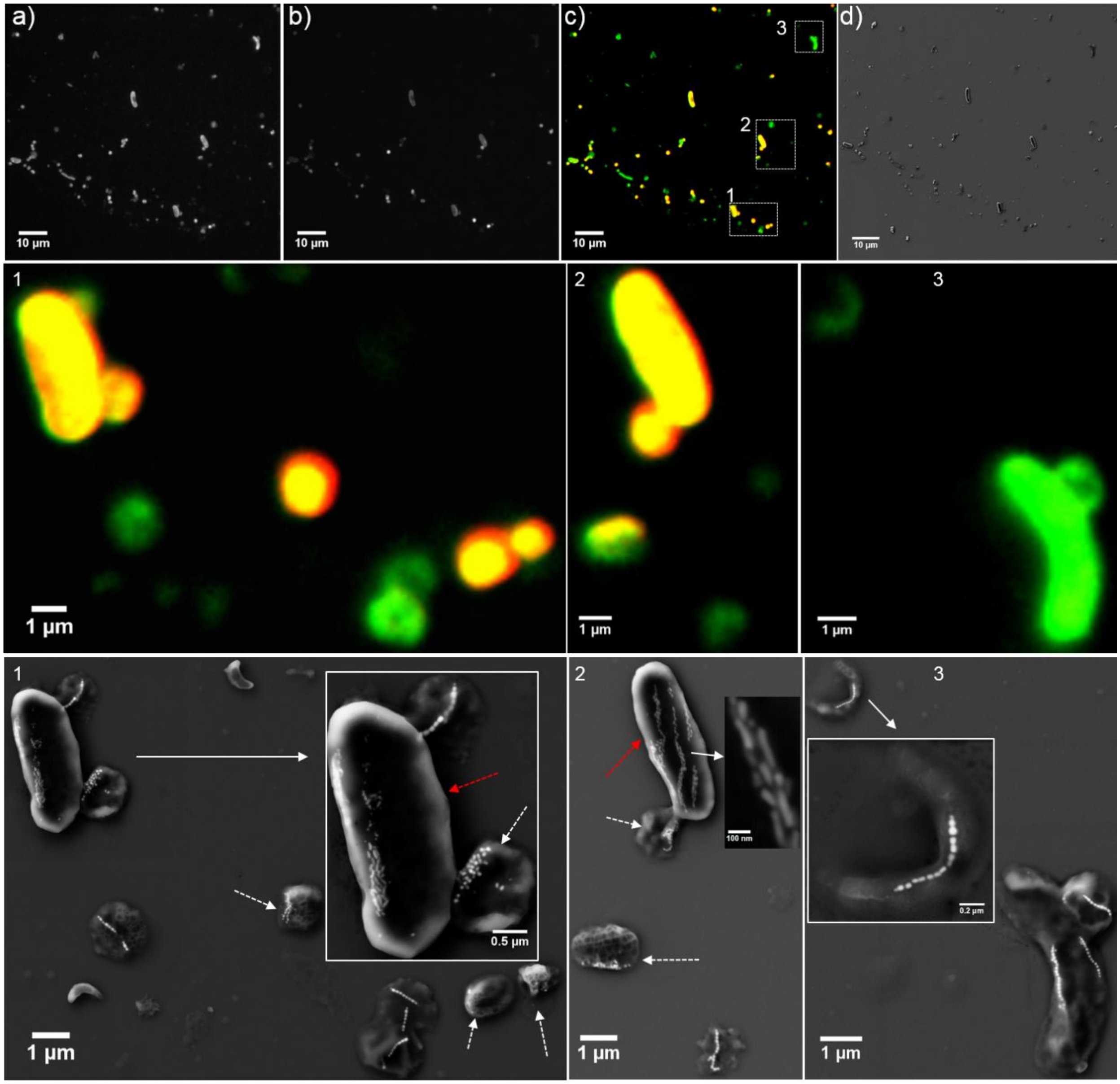

FIG S9 Coupled FISH-SEM identification of uncultured MTB in freshwater sediments collected from Lake Miyun in north Beijing in China based on *in situ* hybridization with the *Desulfobacteriaceae*-specific probe SRB385Db. The same microscopic field is shown following by *in situ* hybridization with the 5'-FAM-labeled universal bacterial probe EUB338 (green) (a) and the 5'-Cy3-labeled *Desulfobacteriaceae*-specific probe SRB385Db (red) (b), and their overlapped fluorescence microscopy image (c). (d) Low-magnification SEM image of the same microscopic field of image c. The second lines show close-ups of fluorescence microscopy images indicated in image c by white dashed squares and numbers. The third lines show high-magnification SEM images of the same microscopic areas to the second line. Magnetotactic *Nitrospirae* strain MYR-1 and one group of magnetotactic cocci which arrange their magnetosomes in dispersed aggregates or clusters were fluorescently labeled by the SRB385Db probe due to its outgroup hits.

Supplementary Material-2

HRTEM images and morphology modelling of SHHR-1 magnetosomes

Single-cell resolution study of uncultured magnetotactic bacteria via fluorescence-coupled electron microscopy

Jinhua Li, Heng Zhang, Nicolas Menguy, Karim Benzerara, Fuxian Wang, Xiaoting Lin, Zhibao Chen, Yongxin Pan

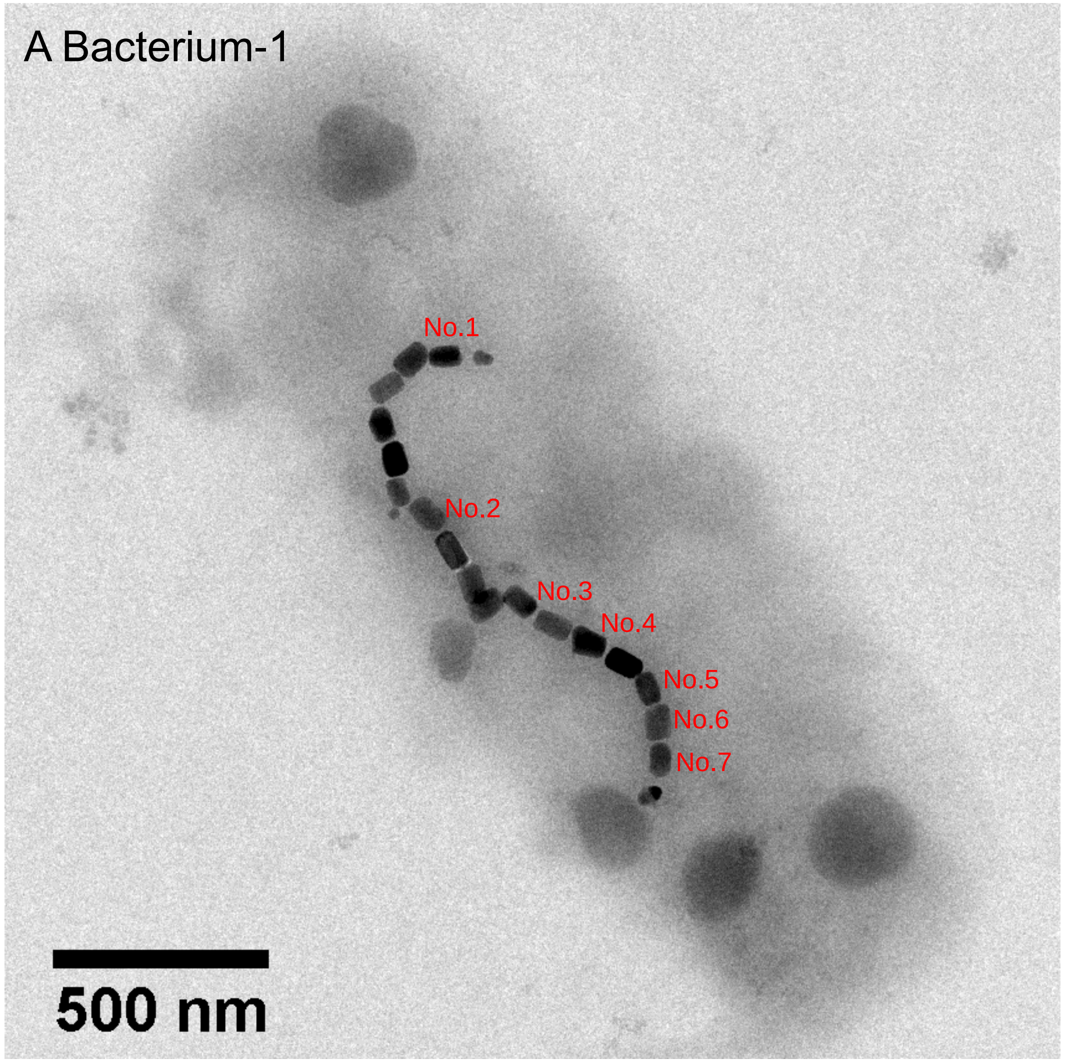
A Bacterium-1
No.1
No.2
No.3
No.4
No.5
No.6
No.7
500 nm

Particle No.	HRTEM image	FFT pattern	Stereographic projection	Morphology model
No.1	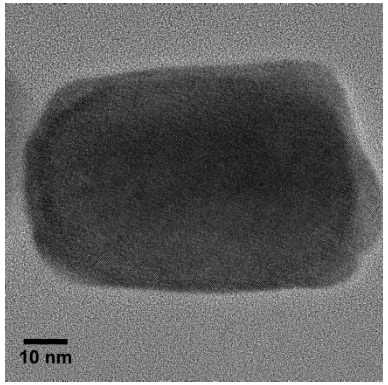	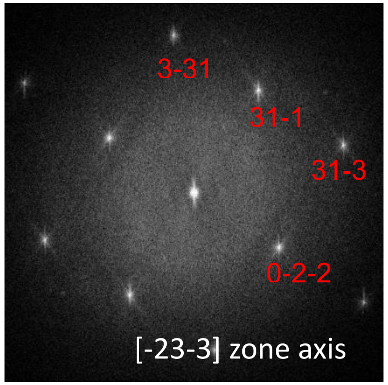	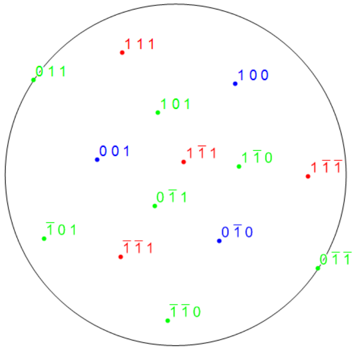	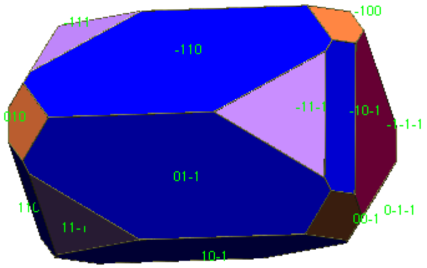
No.2	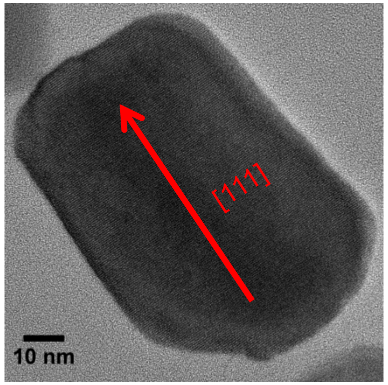	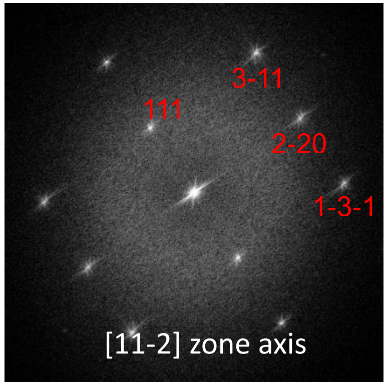	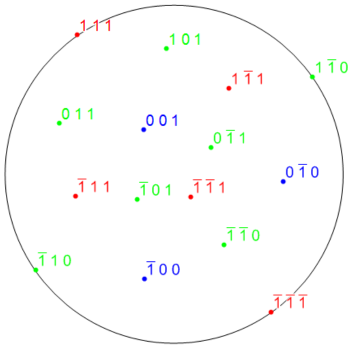	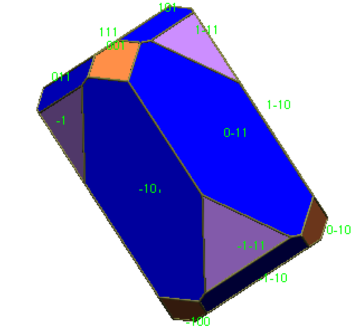
No.3	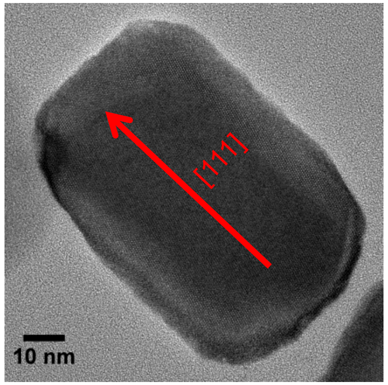	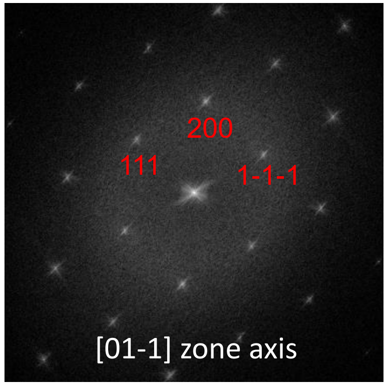	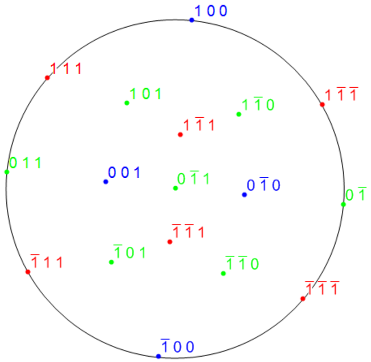	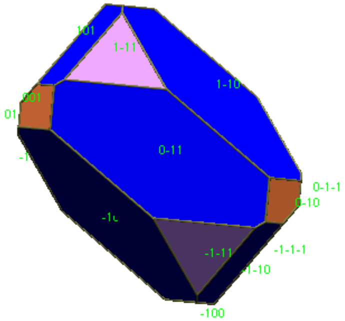

No.4	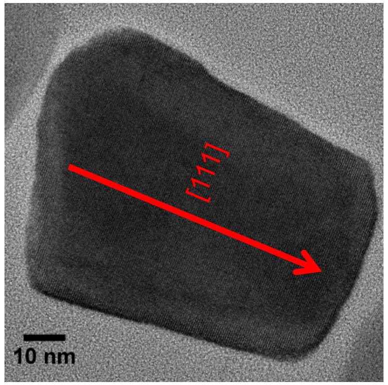 [111] 10 nm	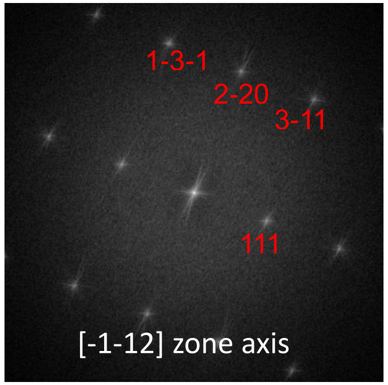 1-3-1 2-20 3-11 111 [-1-12] zone axis	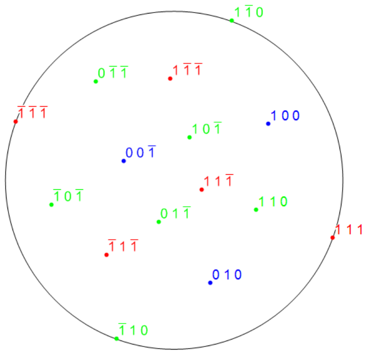	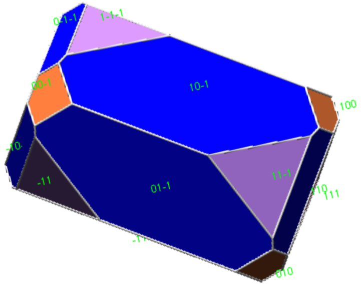
No.5	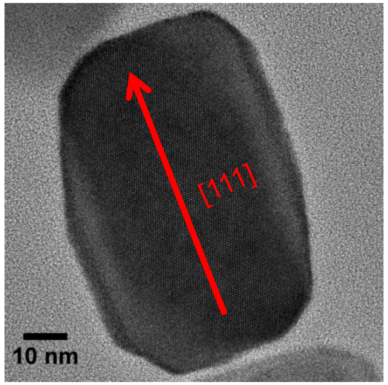 [111] 10 nm	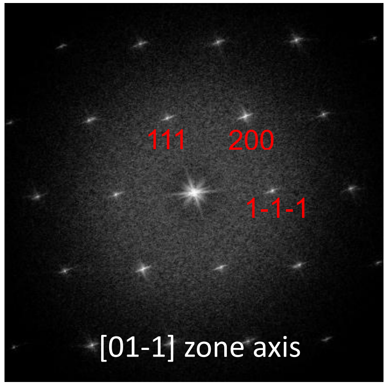 111 200 1-1-1 [01-1] zone axis	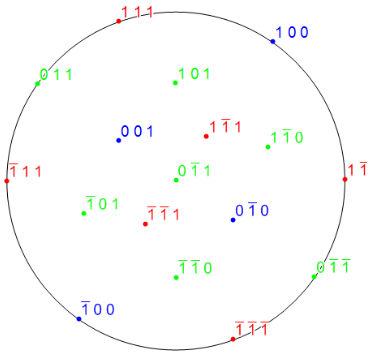	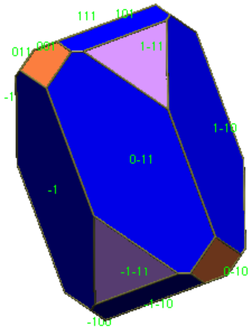
No.6	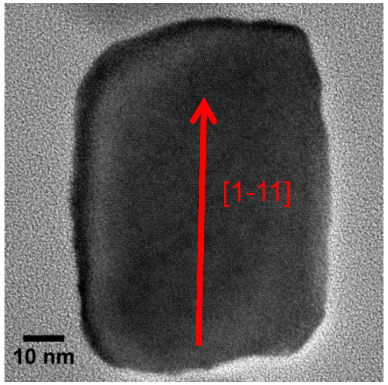 [1-11] 10 nm	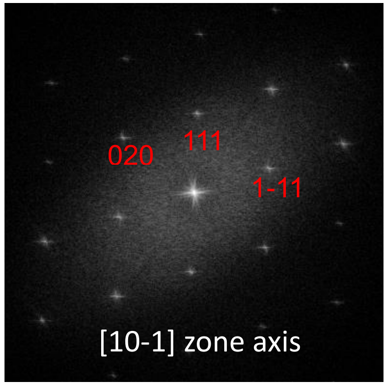 020 111 1-11 [10-1] zone axis	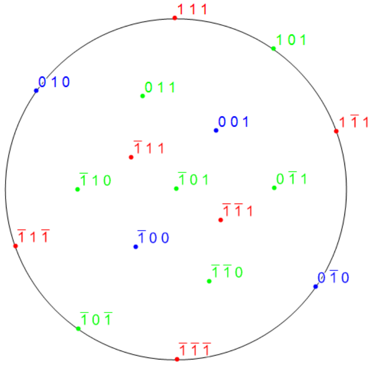	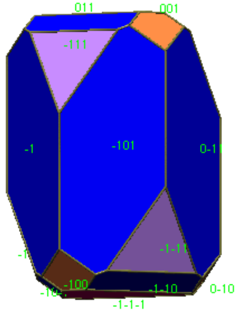

No.7	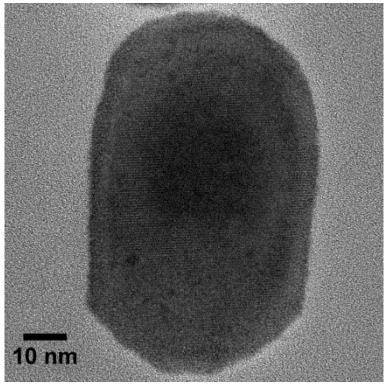	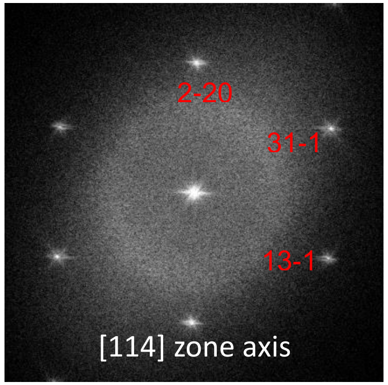	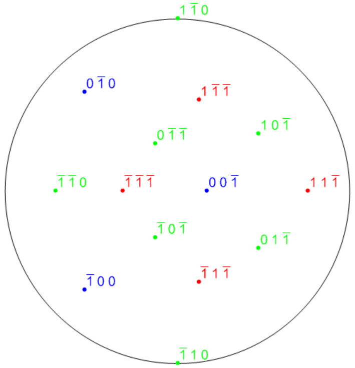	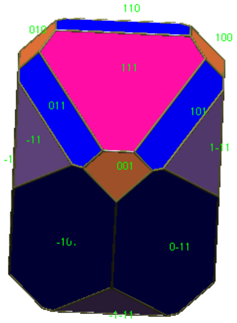

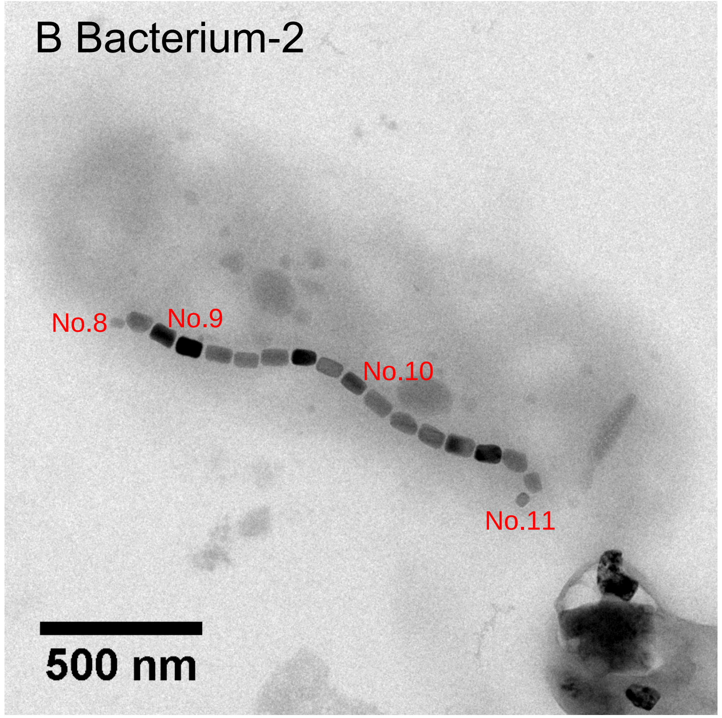
B Bacterium-2
No.8
No.9
No.10
No.11
500 nm

Particle No.	HRTEM image	FFT pattern	Stereographic projection	Morphology model
No.8	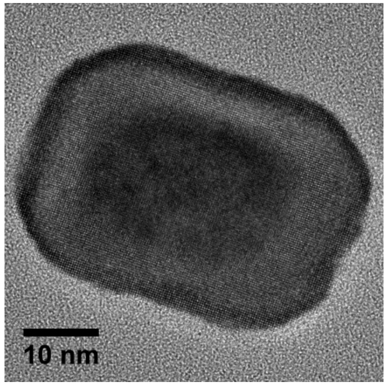	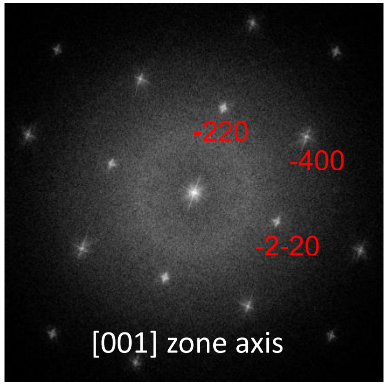	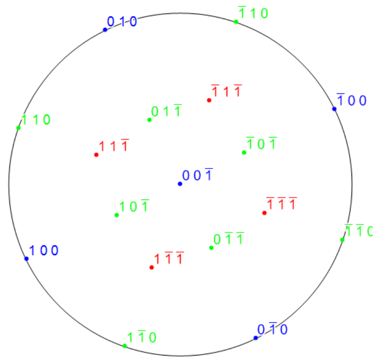	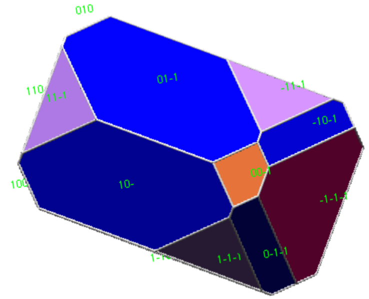
No.9	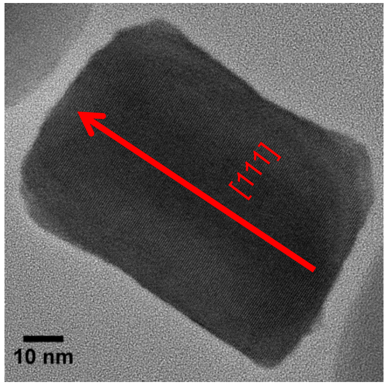	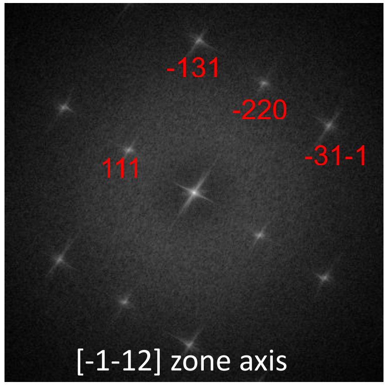	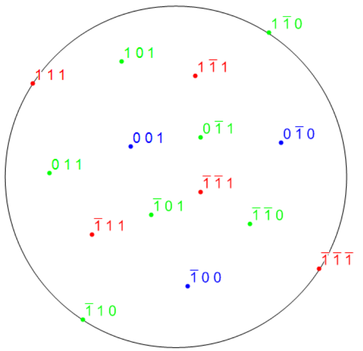	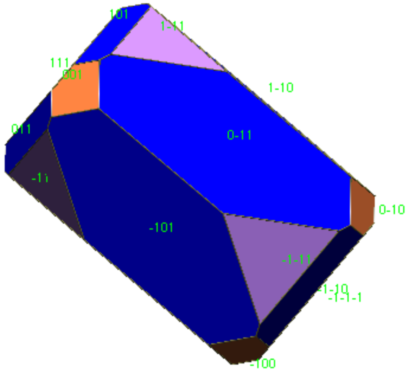
No.10	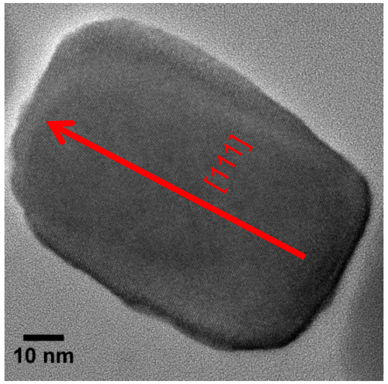	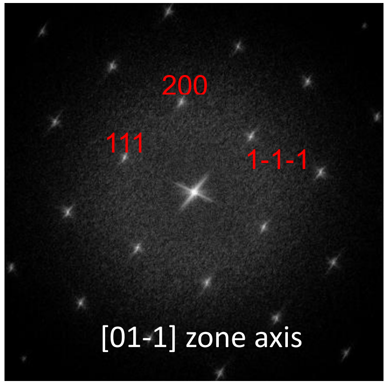	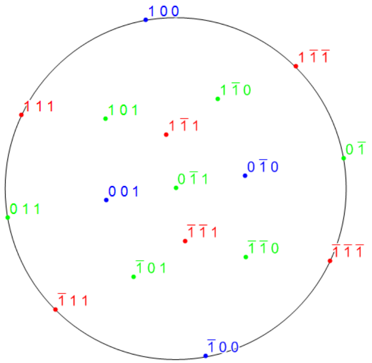	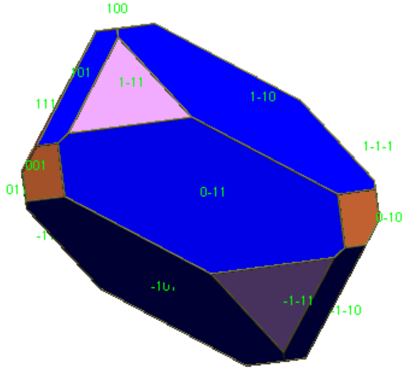

No.11	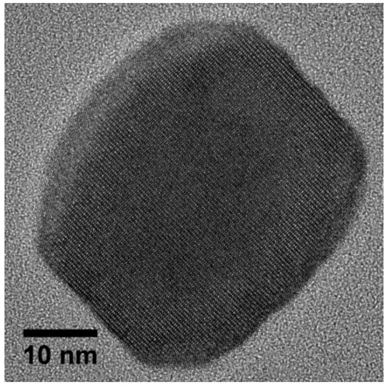	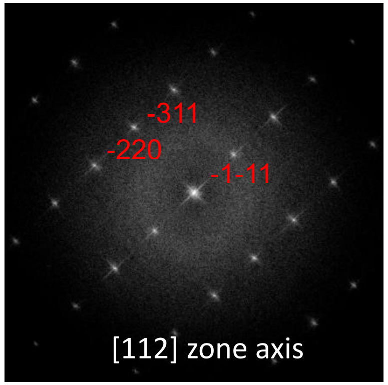	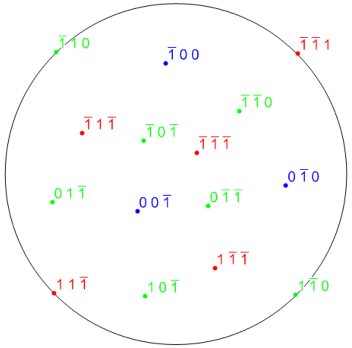	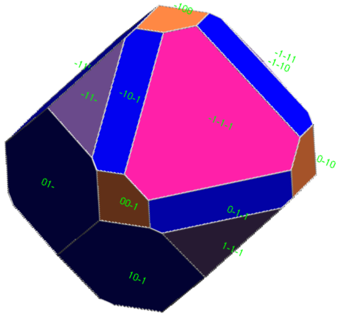
